# Assimilation of Satellite-Based Snow Cover and Freeze/Thaw Observations Over High Mountain Asia

**DOI:** 10.3389/feart.2019.00115

**Published:** 2019-05-22

**Authors:** Yuan Xue, Paul R. Houser, Viviana Maggioni, Yiwen Mei, Sujay V. Kumar, Yeosang Yoon

**Affiliations:** 1 George Mason University, Fairfax, VA, United States; 2 Hydrological Sciences Laboratory, NASA/GSFC, Greenbelt, MD, United States; 3 Science Applications International Corporation, McLean, VA, United States

**Keywords:** snow mass, soil temperature, surface temperature, data assimilation, High Mountain Asia

## Abstract

Toward qualifying hydrologic changes in the High Mountain Asia (HMA) region, this study explores the use of a hyper-resolution (1 km) land data assimilation (DA) framework developed within the NASA Land Information System using the Noah Multi-parameterization Land Surface Model (Noah-MP) forced by the meteorological boundary conditions from Modern-Era Retrospective analysis for Research and Applications, Version 2 data. Two different sets of DA experiments are conducted: (1) the assimilation of a satellite-derived snow cover map (MOD10A1) and (2) the assimilation of the NASA MEaSUREs landscape freeze/thaw product from 2007 to 2008. The performance of the snow cover assimilation is evaluated via comparisons with available remote sensing-based snow water equivalent product and ground-based snow depth measurements. For example, in the comparison against ground-based snow depth measurements, the majority of the stations (13 of 14) show slightly improved goodness-of-fit statistics as a result of the snow DA, but only four are statistically significant. In addition, comparisons to the satellite-based land surface temperature products (MOD11A1 and MYD11A1) show that freeze/thaw DA yields improvements (at certain grid cells) of up to 0.58 K in the root-mean-square error (RMSE) and 0.77 K in the absolute bias (relative to model-only simulations). In the comparison against three ground-based soil temperature measurements along the Himalayas, the bias and the RMSE in the 0–10 cm soil temperature are reduced (on average) by 10 and 7%, respectively. The improvements in the top layer of soil estimates also propagate through the deeper soil layers, where the bias and the RMSE in the 10–40 cm soil temperature are reduced (on average) by 9 and 6%, respectively. However, no statistically significant skill differences are observed for the freeze/thaw DA system in the comparisons against ground-based surface temperature measurements at mid-to-low altitude. Therefore, the two proposed DA schemes show the potential of improving the predictability of snow mass, surface temperature, and soil temperature states across HMA, but more ground-based measurements are still required, especially at high-altitudes, in order to document a more statistically significant improvement as a result of the two DA schemes.

## 1. INTRODUCTION

**FIGURE 1 |:**
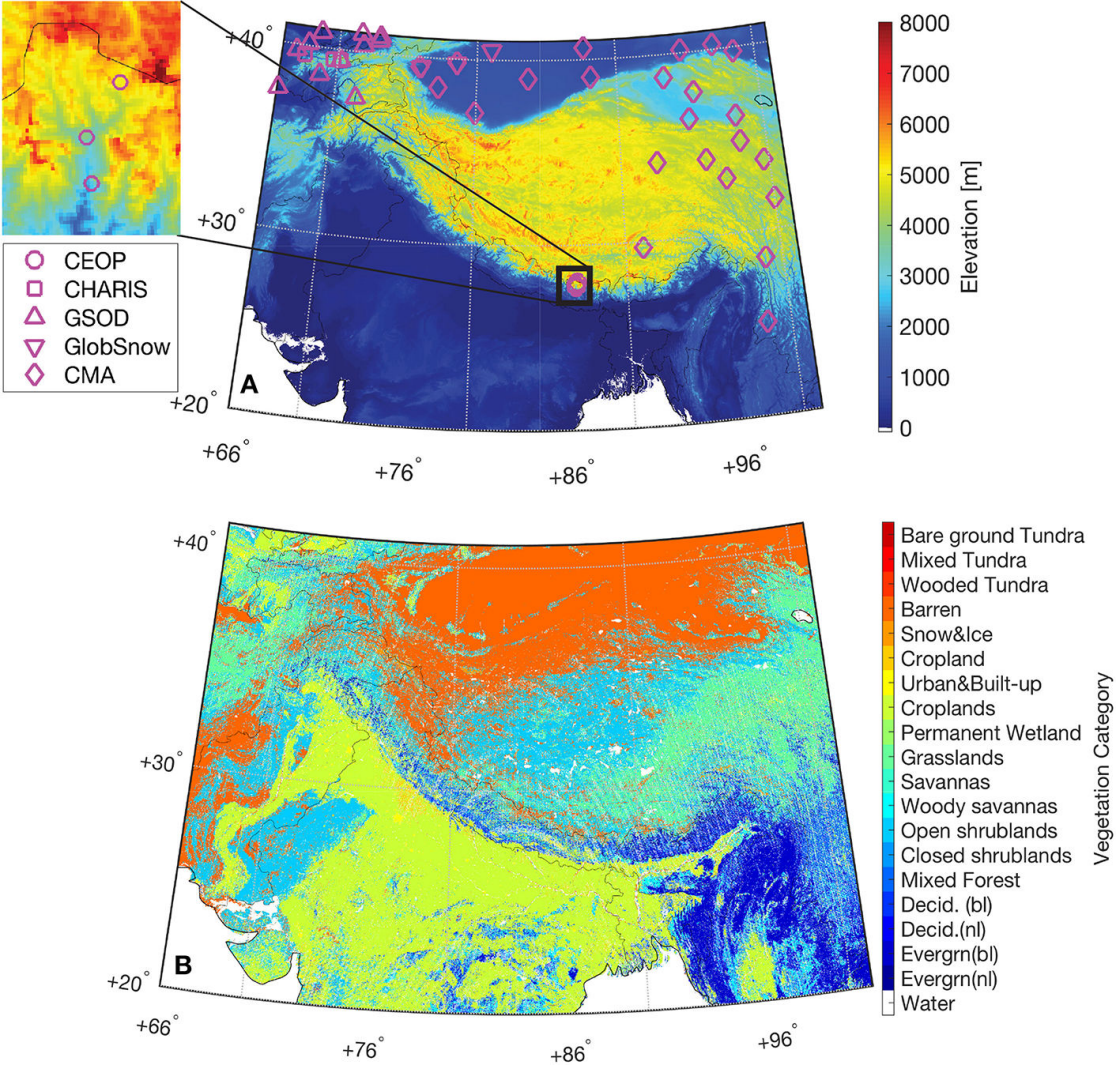
**(A)** Reprocessed Shuttle Radar Topography Mission elevation map on the 0.01° model grid. Grid cells used during assimilation evaluations are marked in magenta. Again, GlobSnow is a product, rather than *in-situ* measurements, but we utilize GlobSnow product in a point-scale manner as discussed in section 4. **(B)** Reprocessed National Centers for Environmental Prediction modified International Geosphere-Biosphere Programme 20-category global vegetation class map, where “Decid.” represents “deciduous trees,” “Evergrn” represents “evergreen trees,” “bl” represents “broadleaf,” and “nl” represents “needleleaf”.

High Mountain Asia (HMA) is a landscape of tundra, enormous glaciers, and alpine lakes, in addition to being a storehouse of freshwater central to the well-being of more than one billion people across Asia (Immerzeel et al., 2010). Snow and glacier melt are important hydrologic processes in HMA (Immerzeel et al., 2009, 2010), and changes in surface temperature are expected to seriously affect the surface melt characteristics (Barnett et al., 2005; Immerzeel et al., 2010) as well as subsurface conditions, such as permafrost (Wu et al., 2013). Quantifying changes to this fragile environment is of utmost importance to protect, restore, and promote sustainable use of the HMA ecosystem, including—but not limited to—water resource management (Immerzeel and Bierkens, 2012) and agricultural activities (Immerzeel et al., 2010; Qiu, 2016). However, the process of developing a better understanding of the HMA ecosystem faces many challenges, especially in the context of climate change (Liu and Chen, 2000; Xu et al., 2008; Yang et al., 2014). For example, the availability of *in-situ* surface measurements for hydrologic, weather, and climate studies in this complex area is scarce, particularly at relatively high altitudes (see Figure 1A). In addition to the lack of dense and stable *in-situ* hydrometeorological measurement networks, high variability in regional weather conditions triggered by the complex topography further complicates the characterization of land processes in HMA (Salzmann et al., 2007). Therefore, a comprehensive knowledge of the regional spatiotemporal variability in the HMA environment might only be achieved by applying advanced modeling techniques, remote sensing products, and data assimilation (DA) methods at relatively high spatial and temporal resolutions.

The Community Noah Land Surface Model with Multi-Parameterization Options (Noah-MP), has been developed and used to simulate land-atmosphere energy, water, and carbon exchanges, as well as key hydrologic states, such as surface runoff, soil moisture, snow depth, snow water equivalent (SWE), and terrestrial water storage at local or basin scales (mainly) over the Continental United States (Niu et al., 2011; Yang et al., 2011; Cai et al., 2014; Chen et al., 2014; Ma et al., 2017). However, few studies have been conducted to rigorously assess the Noah-MP model performance over HMA, particularly across the complex Tibetan Plateau terrain (Zhang et al., 2016), and the majority of these studies (e.g., Gao et al., 2015; Zheng et al., 2015; Zhang et al., 2016) only focus on assessing the effects of new representations of a specific physical process on the improvements of the model’s performance at local scales (Ma et al., 2017). Therefore, it is necessary to evaluate key modeled states, such as snow depth, SWE, surface temperature, and soil temperature estimates in a more systematic manner across the entire HMA.

Land data assimilation systems (LDASs) can optimally merge information from satellite-derived observations and land surface models (usually uncoupled from an atmospheric model) at regional, continental, and global scales (Rodell et al., 2004). LDASs are intended to construct quality-controlled and spatially and temporally consistent land surface datasets from the best available observations and model outputs to support hydrological modeling activities (Mitchell et al., 2004). The ultimate goal of developing such an assimilation framework is to yield a merged state of estimate that is superior to either the observations or model alone (Reichle, 2008). Previous studies found that snow mass and soil moisture modeling performance can be improved through rule-based direct assimilation of (binary) remotely-sensed snow cover (Rodell and Houser, 2004; Zaitchik and Rodell, 2009; Arsenault et al., 2013), and landscape freeze/thaw observations (Farhadi et al., 2015), respectively. The land surface models used in the three aforementioned studies are the Mosaic (in Rodell and Houser, 2004) applied to Continental United States, the Community Land Model (version 2.0) (in Arsenault et al., 2013) applied to Washington and Colorado, United States, and the NASA Catchment Land Surface Model (in Farhadi et al., 2015) applied to North America between 45°N and 55°N and 90°E and 110°E. In the HMA region, few studies showed the potential of LDASs for improving surface soil moisture and skin temperature states by merging remotely sensed observations (e.g., passive microwave brightness temperature observations at relatively coarse spatial resolutions) into land surface models across the Tibetan Plateau (Rasmy et al., 2011; Lu et al., 2012). Based on the relatively encouraging performance of the LDASs investigated in previous studies, this study is intended to integrate the state-of-the-art, remotely sensed snow and freeze/thaw products at relatively fine spatial resolutions into the Noah-MP model to further improve snow- and temperature-related estimates across HMA.

Snow- and land surface-related estimates can be generated from a land surface model at a desired spatial scale. However, they are subject to errors arising from imperfect model parameterizations as well as errors in the boundary conditions used to drive the model. On the other hand, satellite-derived observations (retrievals) are also imperfect due to the instrumentation accuracy, sensor applicability, and retrieval algorithm development assumptions. For example, snow cover extent can be derived from optical (i.e., visible to near-infrared wavelength) satellite sensors at a relatively high spatial resolution [e.g., the Moderate Resolution Imaging Spectroradiometer (MODIS)-derived snow cover extent at 500 m]. The accuracy of these snow cover products is often impacted by atmospheric conditions (e.g., cloud cover). In addition, satellite-based retrievals of snow mass at global scale are available from polar-orbiting platforms carrying microwave sensors, such as the Advanced Microwave Scanning Radiometer–Earth Observing System (AMSR-E)-based SWE product (Tedesco and Narvekar, 2010). This product is available at a spatial resolution of 25 km, but has been reported to yield a high degree of uncertainty over densely-vegetated areas and regions with relatively deep snowpack due to the sensor’s saturation depth, snow grain size evolution, and/or inaccurate representation of snow density in the retrieval algorithm (Foster et al., 1997; Tedesco and Narvekar, 2010). Comparatively, snow cover observations are more preferable for use during assimilation than satellite-based SWE retrievals in this study because (1) satellite-based SWE retrievals are too coarse in spatial resolution, (2) SWE retrievals are prone to relatively large uncertainties, and (3) passive microwave sensors typically do not resolve snow conditions well in mountainous areas. The relatively poor predictability of the AMSR-E based SWE product is also partially demonstrated by Dai et al. (2012) as applied in Xinjiang, China. Without appropriate corrections in the bias of the satellite-based snow mass retrievals, very little improvements—or even degradations—are likely to occur during the assimilation phase according to previous studies (Andreadis and Lettenmaier, 2006; De Lannoy et al., 2012; Liu et al., 2013).

Similarly, the relatively long record of satellite-based retrievals of land surface conditions, such as land surface temperatures and freeze/thaw states, provides LDASs with a considerable number of possibilities to improve hydrological and biospheric processes in weather and climate models. Satellite-based land surface temperature retrievals can be obtained from a variety of polar-orbiting and geostationary platforms carrying infrared (e.g., Wan and Li, 1997; Jin, 2004) and microwave (e.g., Holmes et al., 2009) sensors. Infrared land surface temperature retrievals are largely impacted by weather conditions (i.e., cloud cover, water vapor amount, and aerosols), whereas the accuracy of microwave land surface temperature retrievals are often associated with surface type determination and surface emissivity estimates. On the other hand, satellite-based freeze/thaw states can be obtained from radiometer (e.g., Bateni et al., 2013) and scatterometer (e.g., Bartsch et al., 2007) measurements at various frequencies along the microwave spectrum. Previous studies showed that satellite-based retrievals of land surface temperatures typically exhibit different mean values and variabilities from model estimates (Jin, 2004; Reichle et al., 2010) and/or *in-situ* measurements (Jin et al., 1997) due to differences between satellite overpass times (along with look angles), model output times, and ground-based measurement times. Comparatively, satellite-based freeze/thaw state assimilation is more preferable than that of satellite-based surface temperature retrievals (e.g., from MODIS), partly because the microwave-based MEaSUREs freeze/thaw product is less affected by cloud contamination than the infrared-based MODIS surface temperature product. In addition, the assimilation of satellite-based surface temperature retrievals generally requires prior scaling and/or bias adjustment during the assimilation process (Reichle et al., 2010) because of different climatologies of model estimates and satellite-based retrievals (see Figure 6 for an example of Noah-MP estimates and MODIS surface temperature products). To avoid subjective assumptions of what climatology is more correct, we chose not to assimilate the MODIS surface temperature product.

In this paper, we systematically evaluate the ability of the baseline Noah-MP model along with two data assimilation schemes to simulate surface temperature, soil temperature, snow depth, and SWE states in HMA. Specifically, this work aims to (1) assess the performance of snow depth and SWE estimates simulated by Noah-MP (with and without snow assimilation) and (2) assess the performance of surface temperature and soil temperature profile estimates simulated by Noah-MP (with and without freeze/thaw states assimilation).

## 2. STUDY AREA, MODEL, AND DATASETS

### 2.1. Study Area and Noah-MP Land Surface Model

The study domain is the HMA region bounded between 20°N and 41°N and 66°E and 101°E (see Figure 1). The forward (prognostic) model used in this study is the Noah-MP (version 3.6; Niu et al., 2011; Yang et al., 2011) forced by meteorological fields from Modern-Era Retrospective analysis for Research and Applications, Version 2 (MERRA-2; Gelaro et al., 2017). The Noah-MP model is integrated forward in time at a time step of 15 min from 1 September 2007 to 31 August 2008 on a regular 0.01° spatial grid using the NASA Land Information System (LIS) version 7.2 (Kumar et al., 2006). Noah-MP outputs are generated on a daily-averaged basis, which is consistent with the temporal resolution of the majority of the measurements or products used during the evaluation phase. The model is spun up, reaching quasi-equilibrium of both surface and subsurface temperature states, by looping eight times through the 1-year period from 1 January 2001 to 1 January 2002 (see section 5.1 for details) and then once through the 5.75-year period from 1 January 2002 to 31 August 2007.

Within LIS, the MERRA-2 forcing fields (i.e., air temperature, specific humidity, downward longwave flux, downward shortwave flux, zonal wind, meridional wind, surface pressure, total pressure, total precipitation, and convective precipitation), originally with an hourly temporal resolution and a 0.5° × 0.625° spatial resolution, are spatially interpolated using bilinear interpolation onto the 0.01° model grid and temporally interpolated using linear interpolation onto the same model time step. No additional physically-based downscaling procedure (e.g., temperature or humidity lapse rate corrections) is applied to the atmospheric forcing variables in this study. An advanced downscaling framework (https://eospso.gsfc.nasa.gov/sites/default/files/eo_pdfs/Mar_Apr_2018_color%20508_0.pdf) will be included in the future to evaluate the impact of high-resolution atmospheric forcings on hydrologic modeling. It is worth noting that the MERRA-2 product provides both uncorrected and corrected (i.e., gauge-corrected) precipitation fields. This study utilizes the uncorrected precipitation field from MERRA-2 because the corrected precipitation field may inherit a dry bias from the gauge measurements according to Ghatak et al. (2018) based on their findings in South Asia.

The static input data for Noah-MP are obtained from the National Center for Atmospheric Research/Research Application Laboratory website (https://ral.ucar.edu/solutions/products/noah-multiparameterization-land-surface-model-noah-mp-lsm), which are preprocessed (or re-gridded) onto the same 0.01° model grid using the NASA Land surface Data Toolkit (LDT) public release of version 7.2 (Arsenault et al., 2018). The soil texture types are aggregated from the 30-s, 16-category hybrid State Soil Geographic Database/Food and Agriculture Organization 0–30 cm top-soil texture. The vegetation (land-use) types are obtained from the 1 km, National Centers for Environmental Prediction modified International Geosphere-Biosphere Programme 20-category global vegetation class map (see Figure 1B). The bottom boundary layer conditions for Noah-MP soil models are obtained from the 1-degree annual 2-m air temperature, which are processed from the European Centre for Medium-Range Weather Forecasts model analysis. The monthly climatological green vegetation fraction, monthly climatological surface albedo, and maximum albedo over snow covered area are obtained from the National Centers for Environmental Prediction for the Americas/Global Energy and Water Cycle Experiment America Prediction Project.

The Noah-MP is developed based on the original Noah land surface model, with a number of enhancements including (1) the addition of improved physical processes [e.g., separation of the vegetation canopy from the ground surface (section 2.1.2), (2) the inclusion of a multi-layer snow model (section 2.1.1)] (Dickinson et al., 1998; Yang and Niu, 2003; Niu et al., 2007), and (3) the addition of multiparameterization options (Niu et al., 2011; Yang et al., 2011; Cai et al., 2016), which allow a user to configure the model with different options. Table 1 summarizes all Noah-MP options and parameters used in this study.

#### 2.1.1. Noah-MP Snow Physics

Similar to the legacy Noah model, the snow accumulation/ablation parameterizations of the Noah-MP model are based on mass and energy balance in the snowpack. The change in SWE is balanced by the input snowfall, and output snowmelt and snow sublimation (Wang et al., 2010). Snow compaction, melting, and freezing processes are all taken into account via physically-based snow processes (Niu et al., 2011).

Unlike the legacy Noah model, the snowpack can be divided by up to three layers depending on the snow depth in Noah-MP (Niu et al., 2011). When snow depth is <0.025 m, no snow layer exists and the snowpack is combined with the soil layer. When snow depth is between 0.025 and 0.05 m, a single-layer snowpack is formed. When snow depth is in between 0.05 and 0.15 m, a two-layer snowpack is created. When snow depth is above 0.15 m, a three-layer snowpack is formed. In addition, the snow interception routine in the Noah-MP is employed to account for the loading and unloading of snowfall, melting of intercepted snow (e.g., by the vegetation canopy) and refreezing of the meltwater, frost (or sublimation), and dew (or evaporation). The Noah-MP derived snow cover fraction (on the ground) is parameterized as a function of the snow depth, ground roughness lengths, and snow density (Niu et al., 2007, 2011).

Based on the Noah-MP derived snow cover fraction and SWE on the ground, the model grid cells are categorized into three types: (1) snow-covered, (2) snow-free, and (3) undetermined. If the Noah-MP derived snow cover fraction is greater than or equal to 50% and the modeled SWE is greater than 1 mm, the model grid cell is considered as “snow-covered.” On the other hand, if the model derived snow cover fraction is <50% and the modeled SWE is less than or equal to 1 mm, the model grid cell is considered as “snow-free.” All other cases are considered as undetermined model grid cells in terms of the binary snow cover output.

#### 2.1.2. Noah-MP Temperature States

One of the augmentations of Noah-MP with respect to the legacy Noah model is the separation of vegetation canopy (from the ground surface) to account for vegetation effects on surface energy and water balances. Using a “semi-tile” subgrid scheme, the Noah-MP is able to represent land surface heterogeneity appropriately (Niu et al., 2011). The Noah-MP has the structure of a single-layer of canopy cover. The canopy temperature state and the bare ground temperature state are both solved iteratively via the evaluations of the surface energy balance of solar radiation, longwave radiation, sensible heat, latent heat, and ground heat flux (Niu et al., 2011; Ma et al., 2017). The surface temperature in Noah-MP is then diagnosed from the areal-weighted average of the canopy temperature and the bare ground temperature within a model grid cell. In other words, the canopy layer, the bare ground layer, and the diagnosed “combined surface layer” are all associated with zero heat capacities.

layer center (from the ground surface)A four-layer soil column configuration is used in the Noah-MP model (see Table 1). The thicknesses of each soil layer (from top to bottom) are 10, 30, 60, and 100 cm. Using the ground heat flux (at the surface) as the upper boundary, the soil temperatures of the four-layer soil column are solved together through a tri-diagonal matrix of the implicit time scheme with soil thermal diffusivity properties (Niu et al., 2011). Soil temperature values obtained from Noah-MP represent the temperatures at each soil layer center (from the ground surface) at 5, 25, 70, and 150 cm, respectively.

Based on the Noah-MP derived surface temperature, the model grid cells are categorized into three types: (1) frozen, (2) thawed, and (3) undetermined. If the Noah-MP derived surface temperature is greater than or equal to 274.15 K (+1 °C; *T*_*ub*_), the model grid cell is considered as “thawed.” On the other hand, if the model derived surface temperature is ≤ 272.15 K (−1 °C; *T*_*lb*_), the model grid cell is considered as “frozen.” All other cases (i.e., between *T*_*lb*_ and *T*_*ub*_) are considered as undetermined model grid cells in terms of the binary freeze/thaw output. It is important to note that in most studies, 0 °C is considered as the temperature threshold between the frozen and thawed states (e.g., Colliander et al., 2012; Farhadi et al., 2015; see also section 2.2). The upper temperature boundary (+1 °C; also denotes as “T_*ub*_” in Equation 2) and the lower boundary (−1 °C; also denotes as “T_*lb*_” in Equation 1) are used in a similar manner as Farhadi et al. (2015) for binary freeze/thaw categories, which is used to account for the threshold temperature uncertainty associated with the freeze-thaw transition due to landscape heterogeneity and water solute concentration.

### 2.2. Satellite-based Snow Cover and Freeze/Thaw Observations

The satellite-based snow cover observations are obtained from MODIS Snow Cover Daily L3 Global 500-m Grid (MOD10A1, version 6; Hall and Riggs, 2016). Snow cover in this data set consists of a single, best observation of the day for each grid cell selected from the MODIS/Terra Snow Cover 5-Min L2 Swath 500 m data set. Each observation represents the best sensor view of the surface in the grid cell based on solar elevation, distance from nadir, and grid cell coverage (Hall and Riggs, 2016). The daily, binary snow cover maps are then processed from the MOD10A1 product, with ones (i.e., representing snow-covered conditions) and zeros (i.e., representing snow-free conditions) for land pixels, and “no-value” flags for water bodies or indecisive grid cells (e.g., missing data). If the MOD10A1-derived product observes a Normalized Difference Snow Index snow cover percentage greater than 0 and less than or equal to 100, the land grid cell is treated as “snow-covered.” When the index of snow cover percentage = 0, the land pixel is treated as “snow-free.” For all other cases, the “no-value” flags are applied. These binary snow cover maps are subsequently re-gridded onto the 0.01° model grid using the nearest neighbor interpolation, for later use in the snow cover assimilation (SC DA) scheme. That is, the model and observational information are mapped 1:1 spatially where satellite-based observations are coincidental with model grid cells. It is important to note that the MODIS/Aqua Snow Cover product (MYD10A1, version 6; Hall and Riggs, 2016) is not used in this study because 75% (15 out of 20) of the detectors in the Aqua MODIS band 6 (1.628–1.652 mum) failed shortly after launch. The band 6 is important for the Normalized Difference Snow Index computation. Even though an additional quantitative image restoration technique had been developed to restore the missing band 6 signals used in the MYD10A1 production (Riggs et al., 2017), the MOD10A1 product without the extra image restoration process is deemed preferable in this context.

The satellite-based freeze/thaw observations are obtained from the Making Earth System Data Records for Use in Research Environments (MEaSUREs) Northern Hemisphere Polar Equal-Area Scalable Earth Grid 2.0 Daily 6 km Land Freeze/Thaw Status from the AMSR-E and the AMSR-2 (version 1; Kim et al., 2017, 2018). The MEaSUREs product is used here because (1) it is a publicly available product covering the entire HMA, (2) it has a relatively fine spatial resolution, and (3) it yields relatively high spatially-averaged agreement (greater than 80%) among other satellite-based freeze/thaw products when compared to the offline Noah-MP derived estimates (before assimilation; results not shown). The algorithm identifies surface freeze/thaw state changes based on the dynamic relationship between vertically-polarized brightness temperature observations at 36.5 GHz and changes in the aggregate landscape dielectric constant associated with transitions between predominantly frozen and non-frozen conditions with 0 °C being the temperature threshold (Kim et al., 2011).

Both morning (AM) and afternoon (PM) binary freeze/thaw states are employed in this study, with zeros representing the frozen landscape and ones representing the non-frozen (or thawed) landscape. For all other cases (e.g., water bodies or grid cells not significantly affected by cold season constraints), the “no-value” flags are applied. Similar to the reprocessing procedure of the binary snow cover observations, both AM and PM binary freeze/thaw maps are re-gridded onto the 0.01° model grids using the nearest neighbor interpolation for later use in the freeze/thaw assimilation (FT DA) scheme.

## 3. DATA ASSIMILATION METHOD AND EXPERIMENTAL DESIGN

There is a variety of assimilation techniques to choose from, ranging from the direct insertion (DI) method, Kalman filter (or with its variants, such as an ensemble Kalman filter or an extended Kalman filter), particle filter, Kalman smoother (or with its variants, such as an ensemble Kalman smoother), and variational methods (Walker et al., 2003) to different hybrid assimilation methods that combine two or more techniques together. More sophisticated DA methods (e.g., ensemble Kalman filter) might produce more optimal results than the DI method (Arsenault et al., 2013), partly because the latter treats observations being perfect without dynamically analyzing the relationship between model errors and observation errors, as an ensemble Kalman filter would do. However, sophisticated DA approaches, other than DI methods, generally rely on the existence of a continuous relationship between model states and observations (Walker et al., 2003). Both snow cover and freeze/thaw observation maps are binary, which relate in non-continuous, threshold fashion to model states, and therefore, rule-based (a.k.a. DI-based) updating schemes described below are employed for this study. It is important to note that satellite-based snow cover observations (i.e., MOD10A1 used in this study) can also be assimilated as snow cover fraction using an ensemble Kalman filter into the Noah-MP model, without being converted to binary snow cover maps as described in section 2.2. However, the relatively simple DI method (i.e., assimilation of binary snow cover maps) is used in this study because it is (1) not impacted by uncertainties associated with the estimation of SWE using the depletion curve as a function of fraction snow cover through an ensemble Kalman Filter (De Lannoy et al., 2012); (2) computationally efficient; and (3) more capable of removing modeled snowpack than adding snowpack (Rodell and Houser, 2004; Arsenault et al., 2013), especially considering that the uncorrected MERRA-2 precipitation used in this region is likely to be positively biased (Xie et al., 2017; Ghatak et al., 2018).

Proper characterization of errors in a DA system is also very important, in terms of both model and observation errors. In the DI-based DA systems presented in this study, the model errors are taken into consideration by applying more stringent thresholds to key state variables as outlined in section 2.1.2 for surface temperatures and in section 2.1.1 for snow-related states. The observation errors are implicitly included during the re-gridding (or re-projection) processes as outlined in section 2.2, and therefore, no additional observation errors are applied in both DA systems.

### 3.1. Snow Cover Assimilation (SC DA)

An accurate representation of the snow mass (e.g., snow depth and SWE) is important in this region because the meltwater generated from the snowpack accounts for the majority of the water budget, from ~50% in the Indus and Amu Darya basins to ~67% in the Syr Darya, Tarim, and Tibetan Plateau basins (Smith and Bookhagen, 2018). Following Rodell and Houser (2004) and Arsenault et al. (2013), the Noah-MP model assimilates satellite-derived binary snow cover observations. The updates take place daily at 00:00 (UTC). If the model derived and the corresponding MODIS derived snow cover observations agree with each other, or the observations are flagged as “no-value” (see section 2.2), or the model derived snow cover estimates are undetermined (see section 2.1.1), then no updates occur.

If the model indicates a snow-covered grid cell, but the observation indicates snow-free conditions, both SWE and snow depth states are reduced to zeros. If the model indicates a snow-free grid cell, but the observation indicates snow-covered conditions, the modeled SWE during the analysis update step is increased to 5 mm, the snow depth is increased to 0.02 m accordingly, and one layer of snowpack is created forcefully despite the single snow layer threshold of 0.025 m (of snow depth) as discussed in Section 2.1.1, which is then used to initiate snowpack growth as described in Rodell and Houser (2004). All other snow-related states, such as number of snow layers, snow depth distribution profile (as a function of the snow layers), snow temperature profile, snow liquid water content, and snow ice content, are also modified accordingly within the Noah-MP routines.

### 3.2. Freeze/Thaw Assimilation (FT DA)

Land surface temperature plays a key role in governing the surface energy balance. It dictates the longwave radiation emitted by the surface and serves as an “anchor” for the soil temperature profile (Crago and Qualls, 2014). It also serves as an important boundary condition, which influences the latent and sensible heat flux partitions to the atmosphere (Reichle et al., 2010). Furthermore, soil temperature plays a key role in the land surface processes by affecting a series of physical, chemical, and biological processes in the soil, such as water and heat flux (Meng et al., 2017). Following Reichle et al. (2010) and Farhadi et al. (2015), the Noah-MP assimilates the satellite-derived binary freeze/thaw observations on a daily basis. The updates take place twice a day at 01:30 (AM;UTC) and 13:30 (PM;UTC), which corresponds to the AM and PM freeze/thaw observations, respectively. If the model derived and the corresponding freeze/thaw observations agree with each other, or the observations are flagged as “no-value” (see section 2.2), or the model derived freeze/thaw conditions are undetermined (see section 2.1.2), then no updates occur. In addition, it is important to note that model grid cells covered with a significant amount of snowpack (i.e., greater than 50% of the snow cover fraction or greater than 5 cm of the snow depth as simulated by the Noah-MP model) are not being updated during the FT DA due to the limited penetration depth of the 36 GHz brightness temperature channel used in the MEaSUREs detection algorithm.

If the model indicates a frozen grid cell, but the observation indicates thawed condition, the increment (d) during the analysis (update) step is then computed as:

(1) $$d=T_{lb}-T_{\mathrm{surf}}^{-},$$

where *T*_*lb*_ (= −1 °C or 272.15 K) is the lower boundary of the freeze/thaw state using the surface temperature (see section 2.1.2), and $T_{\mathrm{surf}}^{-}$ is the modeled surface temperature before update. Similarly, if the model indicates a thawed grid cell, but the observation indicates frozen condition, the increment is then computed as:

(2) $$d=T_{ub}-T_{\mathrm{surf}}^{-},$$

where *T*_*ub*_ (= +1 °C or 274.15 K) is the upper boundary of the freeze/thaw state using the surface temperature (see section 2.1.2). Under both Equations (1, 2) circumstances, the updating scheme does not change the modeled freeze/thaw conditions dramatically before and after the analysis update in order to avoid completely reverting the modeled surface energy and water balance conditions. The increment is directly applied onto the top layer of soil temperature state, and therefore the top layer of soil temperature during the analysis step is computed as:

(3) $$T_{topsoil}^{+}=T_{topsoil}^{-}+d,$$

where $T_{topsoil}^{-}$ is the top layer of soil temperature before update and the $T_{topsoil}^{+}$ is the top layer of soil temperature after update. We applied the increments directly, and completely (1:1) onto the top layer of soil temperature instead of modifying surface temperature directly. This is because the Noah-MP surface layer is associated with zero heat storage (see section 2.1.2), and applying the DA increments onto the modeled surface temperature state directly will have minimum effects on the model forecast. However, the relative error correlation between the “observed” surface temperature and the FT DA state variable of the top layer of soil temperature is somewhat difficult to characterize within the DI approach, let alone the phase shift between the diurnal cycle of the two aforementioned variables (Reichle et al., 2010). A series of error analyses is performed and it is found that, assuming all forcing errors arise from the air temperature only, the daily-averaged changes in the modeled surface temperature and the daily-averaged changes in the top layer of soil temperature are roughly proportional, which could be approximated with a ratio of 1:1 in terms of the daily-averaged changes. It is acknowledged that FT DA analysis updates are performed at two model time steps (i.e., morning and afternoon time) rather at the daily-averaged basis, so the relatively simple ratio applied here might not be accurate.

## 4. EVALUATION METHODOLOGY AND REFERENCE DATASET

With limited ground-based stations available over such complex terrain, it is well-acknowledged that HMA is a challenging place to conduct evaluations. In this study, the goodness-of-fit statistics of bias, root-mean-square error (RMSE), and correlation coefficient (R) are adopted for evaluating model derived, daily-averaged snow depth, SWE, surface temperature, and soil temperature estimates. Besides, the 95% confidence intervals are also computed for comparison against *in-situ* surface temperature measurements by assuming a student’s t distribution for spatially-averaged statistics of bias, gamma distribution for RMSE, and an asymptotic normal distribution for R after a Fisher Z transformation, considering each grid cell as an independent data point.

### 4.1. Comparison Against *in-situ* Temperature and Snow Depth Measurements

The performances of both open-loop (OL; no assimilation) and DA estimates (both SC DA and FT DA) are evaluated via comparisons against *in-situ* measurements. Model derived estimates (at a spatial resolution of approximately 1 km) are evaluated against the closest colocated ground-based stations.

The *in-situ*, daily-averaged surface temperature measurements are obtained from the Chinese Meteorological Administration (CMA), namely the Dataset of Daily Climate Data From Chinese Surface Stations for Global Exchange (V3.0) (https://data.cma.cn/en/?r=data/detail&dataCode=SURF_CLI_CHN_MUL_DAY_CES_V3.0&keywords=daily). The daily-averaged surface temperature values provided in this dataset are computed by averaging the four measurements taken by platinum resistance thermometers at 02:00, 08:00, 14:00, and 20:00. One CMA station (not shown in Figure 1A) at (22.57°N, 99.94°E) within the HMA region is removed from the comparison because FT DA is identical to OL for this grid cell (i.e., no analysis updates are performed). Therefore, there are in total 23 CMA stations used for FT DA evaluation.

The *in-situ* soil temperature measurements are obtained from the Coordinated Enhanced Observing Period (CEOP) Asia Monsoon project at the Himalayas site (https://www.eol.ucar.edu/projects/ceop/dm/insitu/sites/ceop_ap/). A total of three CEOP stations are available for the FT DA evaluation. Soil temperatures are measured using DLA400 Lsi-Lastem sensors at a time step of an hour (or 20 min), and at depths of 5 and 20 cm (or 15 cm) from the ground surface (depends on the station). Measurements collected at the depth of 5 cm are used to evaluate the model derived estimates for the top layer of soil (0–10 cm). Measurements collected at the depth of 20 cm (or 15 cm) are used to evaluate the model derived estimates for the second layer of soil (10–40 cm). Since no measurements are available at the center of the second layer of soil (i.e., 25 cm), the Inverse Distance Weighting method is applied onto the model estimates to match with the measurement depths. Daily-averaged temperature values are then computed as the temporal mean of the temperatures collected during the 24-h period of the day as a function of the measured depth. In addition, CEOP also provides users with soil temperature measurement flags to help with data quality controls. Therefore, only soil temperature measurements with the “good” CEOP flags are retained and used in the daily-averaged temperature calculations.

The *in-situ*, daily-averaged snow depth measurements are obtained from (1) the Global Summary of the Day (GSOD; https://data.noaa.gov/dataset/dataset/global-surface-summary-of-the-day-gsod) and (2) the Contribution to High Asia Runoff from Ice and Snow (CHARIS) project (http://himatmap.apps.nsidc.org/hma_insitu.html). It is important to note that *in-situ* snow stations with records less than 20 days during the snow season (from December 2007 to March 2008) are not used in the analysis. Both CHARIS and GSOD provide their station elevation information along with the depth measurements. We use station-provided elevation information to compare against the model grid cell elevation obtained from the Shuttle Radar Topography Mission (see Figure 1A). If the absolute elevation difference between the model grid cell and the GSOD station is greater than 100 m, the station is removed from comparison. One GSOD station (not shown in Figure 1A) at (39.29°N, 71.87°E) within the HMA region is removed because the elevation difference is greater than 3,000 m. The pre-examination of the elevation difference is important because disparities in the horizontal support (i.e., *in-situ* station vs. 1 km model grid cell) will be exacerbated by the differences in vertical elevation, especially in such complex terrain for snow estimates. The implementation of the quality control process finally yields three CHARIS stations and 11 GSOD stations during SC DA evaluation.

### 4.2. Comparison Against Reference Satellite-Based SWE and Surface Temperature Products

The satellite-based snow product used during the evaluation process is the European Space Agency Global Snow Monitoring for Climate Research (GlobSnow) SWE (Pulliainen, 2006; Takala et al., 2011). GlobSnow SWE estimates are a Bayesian combination of a semi-empirical snow emission model (Pulliainen and Grandell, 1999), space-borne passive microwave observations, and ground-based snow depth measurements obtained from adjacent weather stations. GlobSnow SWE is provided daily at a 25 km horizontal resolution, limited between latitudes 35° and 85°N across non-mountainous regions. During the evaluation period, 138 days (out of 365 days) of estimates are missing. The majority of the missing days are in June, July, August, and September during the snow-off or very thin snow season.

The quality of the snow reanalysis product (e.g., GlobSnow SWE product used here) depends on the availability of ground-based snow stations used in the production phase, especially for the HMA region with a limited number of ground-based stations (e.g., Toure et al., 2016). Instead of comparing against the regional GlobSnow SWE estimates, the study only extracts qualified pixels with colocated GlobSnow-provided weather stations. It is important to note that only the station coordinates are provided by GlobSnow. No time series of the station measurements and no ancillary station related information (e.g., station elevation) are provided. There are in total nine qualified model grid cells with colocated GlobSnow weather stations. Due to the scarcity of ground-based measurements across HMA used in their product, only one weather station is available per one GlobSnow pixel.

Similar to the strategy adopted in section 4.1, we only compare the model derived estimates obtained from the single model grid cell with colocated GlobSnow weather station against the corresponding GlobSnow SWE estimates. If the model grid cell has an elevation greater than 3,000 m, the cell is removed from the comparison because GlobSnow SWE is not able to represent mountain snowpack conditions. Thus, two model grid cells (markers not shown in Figure 1A) are removed. In addition, four of the remaining seven qualified model grid cells (markers not shown in Figure 1A) are removed because SC DA is identical to OL (i.e., no SC DA updates are performed). Therefore, only three grid cells are used to compare against GlobSnow estimates. Additionally, it is important to note that the Canadian Meteorological Centre derived daily snow depth (or SWE), also a snow reanalysis product, is not used in the evaluation because this reanalysis product does not provide coordinates of the *in-situ* snow observations used within their production phase. The satellite-retrieved AMSR-E SWE product is not used in the evaluation because it has been reported to significantly underestimate SWE (see sections 1, 3.1 for discussions).

The satellite-based surface temperature products used during the evaluation process are the MODIS/Terra Land Surface Temperature Daily L3 Global 1-km Grid (MOD11A1, version 6; Wan et al., 2015) and the MODIS/Aqua Land Surface Temperature Daily L3 Global 1-km Grid (MYD11A1, version 6; Wan et al., 2015). The MODIS instruments on Terra and Aqua image the same area on Earth approximately 3 h apart. The MODIS instrument observes the instantaneous land surface temperature during the satellite overpass times using infrared bands. Cloud-contaminated observations are removed from both products during their production phases (Wan et al., 2015). The median UTCs of satellite overpasses across the HMA area between 2007 and 2008 are approximately 05:49 (MOD11A1 daytime), 16:46 (MOD11A1 nighttime), 08:15 (MYD11A1 daytime), and 20:47 (MYD11A1 nighttime). Both daytime and nighttime land surface temperatures derived from MOD11A1 and MYD11A1 products are re-gridded onto the 0.01° model grid using the nearest neighbor interpolation. Given the availability of both nighttime and daytime land surface maps generated by MOD11A1 and MYD11A1—in total four measurements—several methods exist that can evaluate the model derived estimates. In this study, we use the simple arithmetic mean of all four measurements to approximate the daily-averaged values (see Equation 4) and then to compare with the model derived daily averages.

(4) $$LST_{m}=1/4*\left(LST_{moddy}+LST_{modnt}+LST_{myddy}+LST_{mydnt}\right)$$

where LST_*m*_ is the MODIS derived, daily-averaged land surface temperature. The subscript “moddy” denotes MOD11A1 daytime product, “modnt” denotes MOD11A1 night product, “myddy” denotes MYD11A1 daytime product, and “modnt” denotes MYD11A1 nighttime product. For a single grid cell, four measurements, including daytime MOD11A1, nighttime MOD11A1 as well as daytime MYD11A1, and nighttime MYD11A1 have to present simultaneously in order to calculate the daily-averaged surface temperature; otherwise, a “no-value” flag is applied.

## 5. RESULTS AND DISCUSSIONS

### 5.1. Model Spin-up

In order to allow the model states of interest with longer memories (e.g., deep-soil temperature) to reach quasi-equilibrium, the model must be properly initialized via spinning up. Using the initial conditions given in Table 2, the Noah-MP model is spun up by looping through several integrations with 2001 forcing data. The year of 2001 is used because it is neither too cold nor too warm, neither too dry nor too wet (Ren et al., 2017), which is relatively representative of the recent (e.g., 2000 and beyond) climate with minimized regional annual anomalies in the meteorological forcings (Rodell et al., 2005). The completion of the spin-up procedure is determined by looping through the model repeatedly until all model states of interest reach their equilibrium states. The equilibrium state is defined as all model grid cells in the study region having to meet the requirement set by Equation (5) across the majority (at least 90%) of the days within the year. That is, the relative difference in the model states between consecutive spin-up years cannot exceed 0.1% across 90% of the year. The 0.1% relative difference criteria is adopted based on the method outlined in Rodell et al. (2005) and Cai et al. (2016).

(5) $$\frac{\left|x_{n+1}-x_{n}\right|}{\left|x_{n}\right|}\leq 0.001,$$

where n defines the n-th loop of the year used in the spin-up procedure and x represents the daily average, a single model grid cell based state variable output (i.e., surface temperature, or each layer of the soil temperature). The operator | · | denotes taking the absolute value of the state variable from the n-th loop as well as the absolute value of state variable difference obtained from the n+1 and n-th loop.

When the fourth layer of soil reaches its quasi-equilibrium, the three other soil layers (0–10, 10–40, 40–100 cm) also reach their own quasi-equilibrium states, which is expected. Noah-MP derived surface temperature generally requires 3–5 years for spin-up, and the fourth layer of soil temperature requires 3–8 years for spin-up (not shown). Longer spin-up periods are often witnessed in the Tibetan Plateau, where homogenized initial temperatures (288.0 K) of soil, vegetation, and ground (see Table 2) applied at the spin-up beginning in January significantly deviate from the comparatively cold climate (relative to the rest of HMA, such as Central India) across the Tibetan Plateau. This phenomenon is especially notable across the western extensions of the Himalayas, such as Karakoram, Pamir, and Hindu Kush mountain ranges, where extremely cold weather persists throughout the year.

### 5.2. Assessments of SC DA

**FIGURE 2 |:**
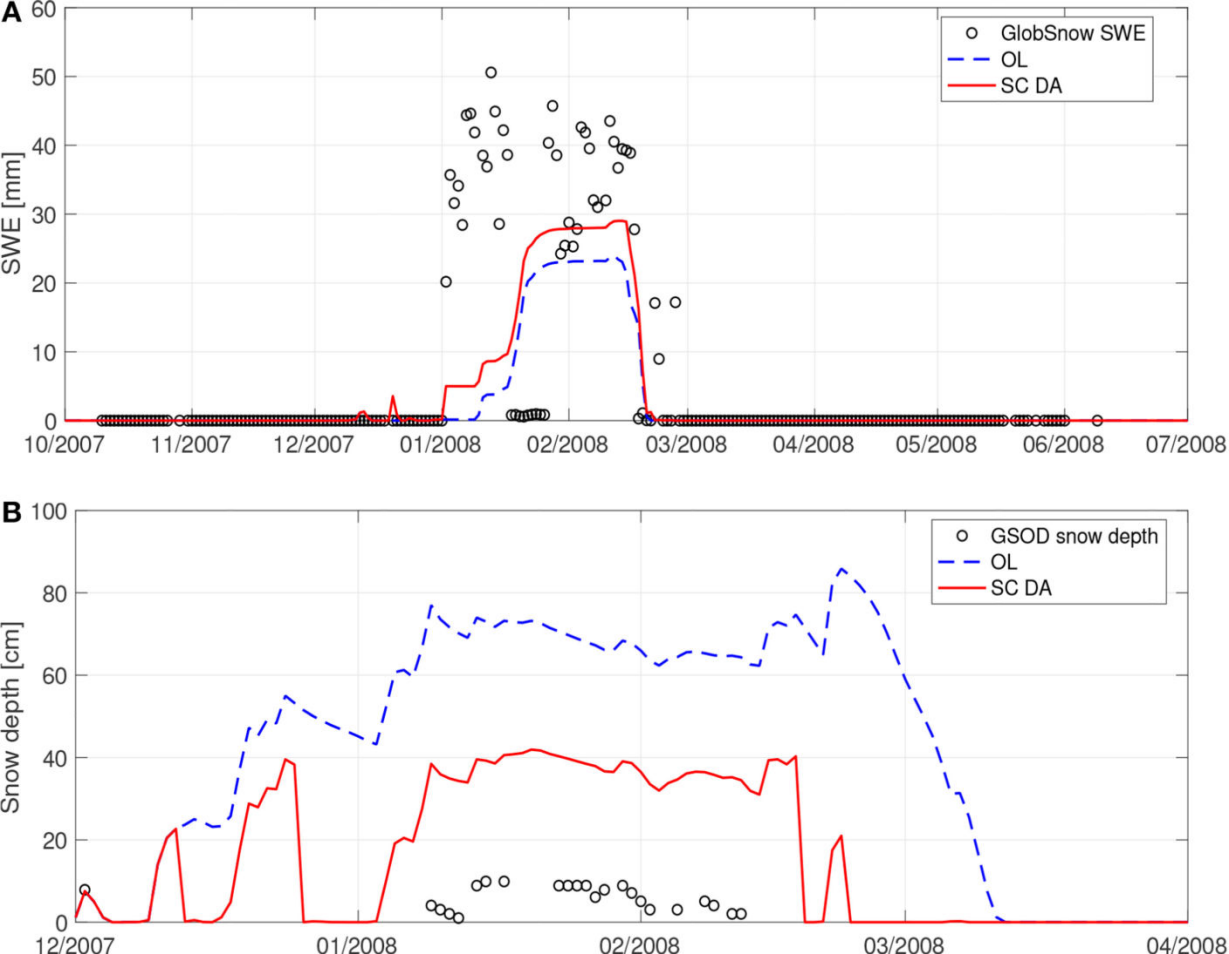
**(A)** Comparison of OL and SC DA derived SWE against GlobSnow SWE estimates at (39.48°N, 76.00°E) between 01 October 2007 and 01 July 2008 (with white gaps representing missing GlobSnow estimates). Time series between September and October, and between July and August are not shown in the Figure because no GlobSnow estimates are available. **(B)** Comparison of OL and SC DA derived snow depth against GSOD snow depth measurements at (38.55°N, 68.83°E) between 1 December 2007 and 1 April 2008 (with white gaps representing missing GSOD measurements). Time series between September and December, and between April and August are not shown in the Figure because no GSOD measurements are available.

Figure 2A shows an example time series of OL and SC DA derived SWE estimates when compared against GlobSnow estimates at a grid cell in an urban area in Xinjiang, China. OL generally underestimates SWE, while SC DA successfully adds some snow on 02 January 2008. Overall, the bias in the model derived SWE estimates is reduced by 35%, the RMSE is reduced by 10%, and the R is increased by 13% as a result of SC DA relative to OL (see Table 3). It is interesting to note that there is a dramatic decrease in the GlobSnow-derived SWE of more than 35 mm on 18 January 2008, when neither OL nor SC DA demonstrates similar behavior. The sharp drop in the SWE might be due to snow plowing activities by the local residents; however, under such circumstances, it is relatively difficult to explain the dramatic, and seemingly unrealistic, subsequent increase in SWE of 40 mm on 27 January 2008. Therefore, it is more likely that GlobSnow derived estimates are prone to higher uncertainty (relative to Noah-MP model estimates) between 18 January 2008 and 27 January 2008 for this grid cell. The erroneous estimates in GlobSnow might arise from the uncertainty in the passive microwave brightness temperature observations used in the algorithm development phase (see section 4.2). Such brightness temperature observation uncertainty might be attributable to urban construction and human activity disturbances in the area (Xiong et al., 2017).

Figure 2B shows an example time series of OL and SC DA derived snow depth estimates in comparisons with GSOD measurements at a grid cell close to Dushanbe airport in Tajikistan. OL generally overestimates snow depth, while SC DA successfully removes some snow since 15 December 2007. Overall, the bias in the model derived snow depth estimates is reduced by 50%, the RMSE is reduced by 50%, and the R is improved from −0.05 to 0.16 as a result of the SC DA relative to OL (see Table 3). Even though SC DA demonstrates significant improvements in snow depth estimates relative to OL, the bias (or RMSE) in the SC DA derived snow depth of 30 cm is still too large. It is possible that the large bias in the model estimates is due to measurement errors in the *in-situ* GSOD dataset. That is, snow depth measurements collected in an open area (i.e., airport) are subject to wind-blown snow redistribution effects that might contain negative biases (Reichle et al., 2011). It is also possible that the large bias in the model estimates might be due to infrequent updates (i.e., removal of snow in this grid cell) in the SC DA along with the overestimation of the precipitation in MERRA-2 (Xie et al., 2017).

Table 3 summarizes the goodness-of-fit statistics, including bias, RMSE, and R, of both OL and SC DA experiments with respect to ground-based CHARIS snow depth measurements, ground-based GSOD snow depth measurements, and the reanalysis product based GlobSnow SWE estimates. Again, the three grid cells shown in Table 3 for GlobSnow SWE comparisons are the ones colocated with GlobSnow-provided weather stations (see section 4.2 for discussions). It is not too surprising to see that the majority of these publicly-available *in-situ* stations were installed at relatively low elevations because highest terrain is too steep, exposed, and/or inaccessible to maintain a snow measuring instrument (Lundquist et al., 2015). It is still encouraging to see that the majority of the *in-situ* stations (13 of 14) installed at relatively low-to-medium altitudes witness improved goodness-of-fit statistics in the snow depth estimates as a result of the SC DA relative to OL. However, only four of the 14 stations witness statistically significant improvements in the SC DA derived evaluation metrics. It is important to note that four ground-based stations, including two CHARIS stations and two GSOD, stations yield biases and/or RMSEs greater than 40 cm in both OL and SC DA experiments. The mean elevation of the four grid cells coinciding with these ground-based stations are approximately 2,500 m according to Figure 1A. Therefore, the large uncertainty in the model estimates might be attributed to the positive bias in the MERRA-2 forcing at relatively high altitudes. It might also be explained by the fact that a single ground-based station is not representative of the snow condition across a 1 by 1 km model grid, especially for the mountain snowpack where the snow is highly variable spatially. During the comparison against GlobSnow SWE estimates, all (three out of three) grid cells show improved (but not statistically significant improved) bias and RMSE in SC DA relative to OL, but only one of them shows slightly improved (but not statistically significant improved) R in SC DA. The exact reason for the degraded statistics in R at some grid cells is unclear since it is relatively difficult to discern the uncertainty of the model derived estimates from the GlobSnow product.

**FIGURE 3 |:**
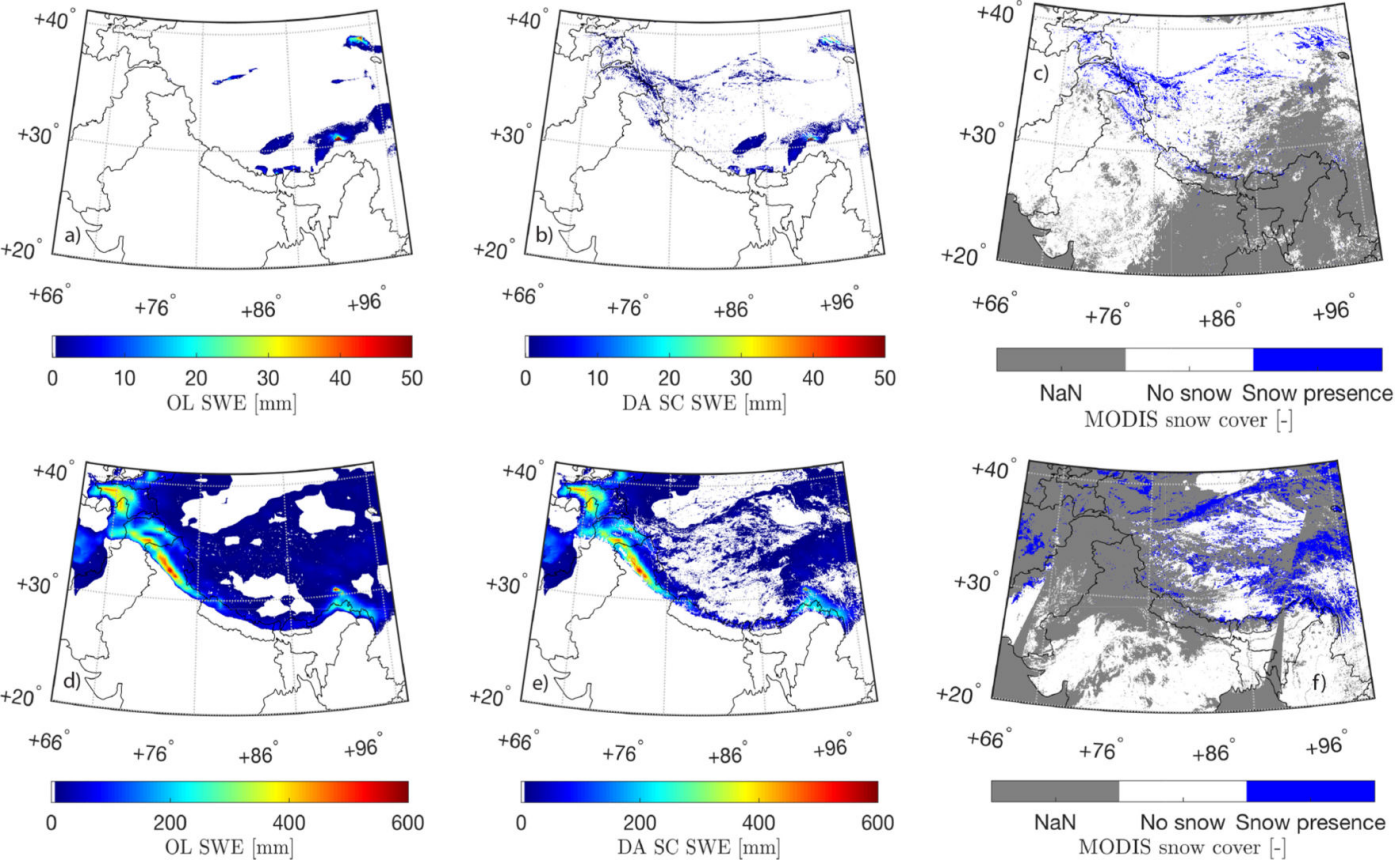
Daily-averaged SWE estimates on 15 September 2007 derived from **(A)** OL and **(B)** SC DA. Daily-averaged SWE estimates on 3 February 2008 derived from **(D)** OL, and **(E)** SC DA. Assimilated MODIS snow cover maps are shown in **(C)** for 15 September 2007 and **(F)** for 3 February 2008, respectively, where “NaN” is the no-value indicator as discussed in section 2.2.

**FIGURE 4 |:**
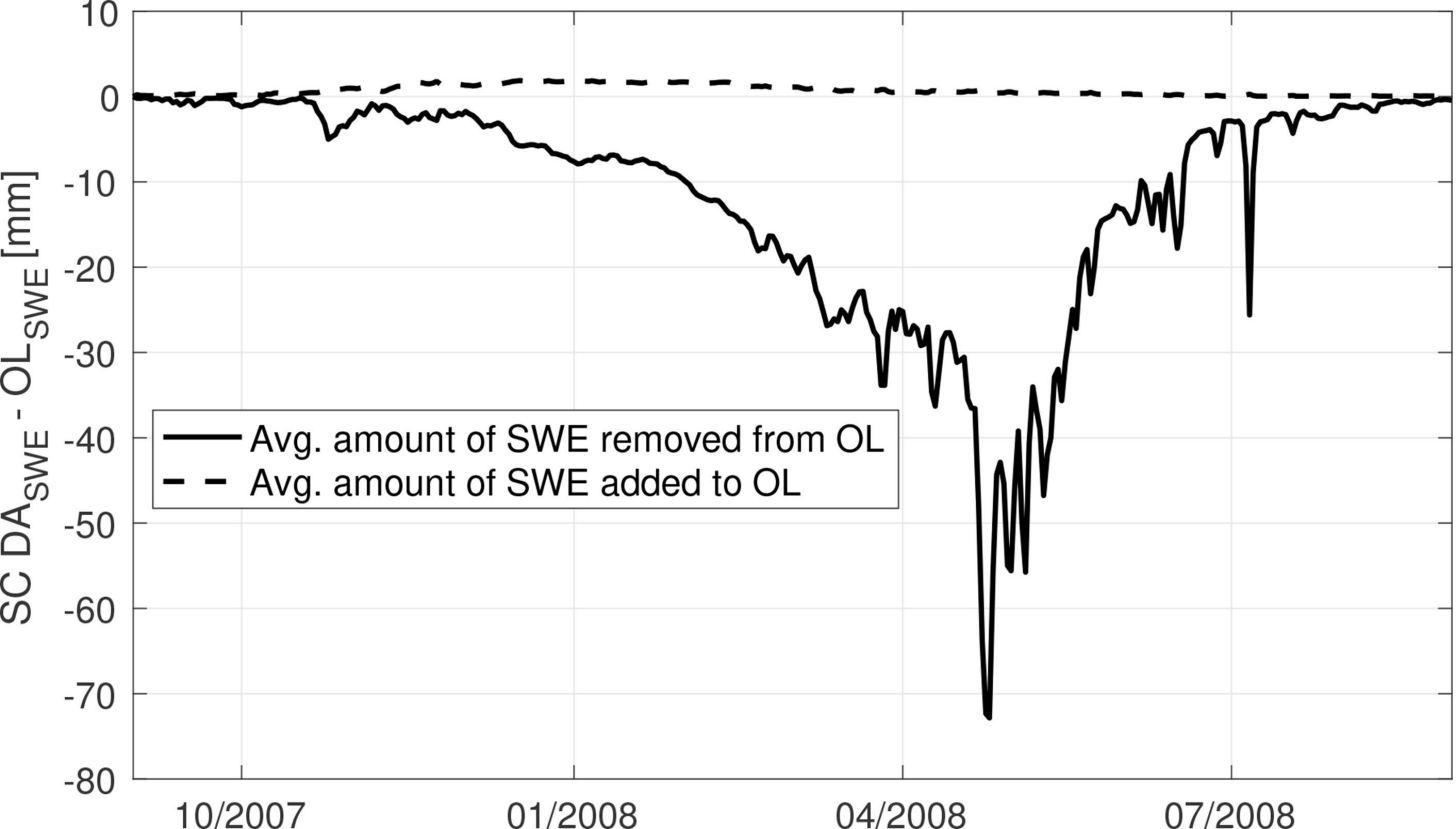
The spatially averaged amount of SWE being removed or added from OL due to SC DA from 2007 to 2008. Grid cells that never go through any SC DA updates are removed from the calculation.

Figure 3 shows daily-averaged SWE estimates derived from OL and SC DA along with assimilated MODIS snow cover maps on 15 September 2007 and 3 February 2008, respectively. At the start of the snow accumulation season in September, slightly more snow is being added to OL model estimates (relative to being removed) as a result of the SC DA along the western extensions of the Himalayas as well as the Kunlun mountain range. It is therefore not surprising to find that compared with OL SWE estimates, the snow estimates pattern derived from SC DA agrees more closely with the MODIS snow cover map as shown in Figure 3C. As the winter season progresses into February, SC DA derived SWE estimates tend to remove more snow from OL especially across the Inner Tibetan Plateau. Similar findings can also be witnessed in Figure 4. The solid line is calculated by averaging all grid cells with lower SC DA derived SWE estimates (relative to OL) as a function of the time, for which the SWE amount difference between SC DA and OL can be used to represent the spatially-averaged amount of SWE being removed from OL due to SC DA. On the other hand, the dashed line is calculated by averaging all grid cells with higher SC DA derived SWE estimates (relative to OL) as a function of the time, for which the SWE amount difference between SC DA and OL can be used to represent the spatially-averaged amount of SWE being added onto OL due to SC DA. Overall SC DA tends to remove more snow from the baseline Noah-MP model, especially during the snow melt season after April, which might be due to (1) the correction of the positive bias in the MERRA-2 derived precipitation (Xie et al., 2017), and/or (2) the capability of DI to remove snow (see section 3). If the precipitation data had a negative bias, we would probably expect less updates during the snow melt period, but perhaps more updates in the peak periods. However, without adequate ground-based stations to evaluate against, it is still difficult to conclude whether SC DA performance is better than OL across the entire Tibetan Plateau.

Based on the evaluations against *in-situ* snow depth measurements and SWE products at point-scale and at relatively low-to-medium altitudes, it is therefore concluded here that SC DA generally performs better than OL in terms of both snow depth and SWE estimates, especially for bias and RMSE statistics. Besides the representativeness issue of the ground-based stations, there exist some limitations with the SC DA direct insertion technique. It is obvious that the improvement (or degradation) magnitudes arising from SC DA are strongly dependent on the number of analysis updates that occurred during the assessment period. Most of the updates take place during early and late snow seasons when observations and modeled estimates do not agree with each other more frequently relative to the other time of the snow season. In other words, the DI-based SC DA is unlikely to initiate a large update in the peak winter. In addition, the increments of SWE and snow depth as applied during the SC DA update phase are somewhat subjective (see section 3.1). For example, Figure 2A shows that an update (increase) of 5 mm of SC DA derived SWE on 02 January 2008 is inadequate to capture the SWE increase in the GlobSnow product. Furthermore, a successful implementation of SC DA is also closely related to an accurate representation of the forcing, especially for precipitation (including snowfall) used in the snow estimation.

### 5.3. Assessments of FT DA

**FIGURE 5 |:**
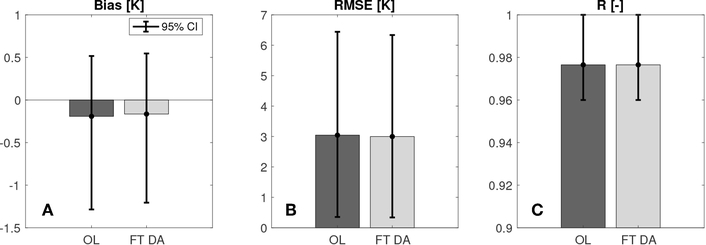
Histogram of the average **(A)** bias, **(B)** RMSE, and **(C)** R computed by comparing OL, and FT DA against CMA ground-based surface temperature measurements. All histograms are supplemented with 95% confidence intervals. It is important to note that **(C)** does not start with 0.

Figure 5 shows the histograms of average bias, RMSE, and R computed by comparing OL and FT DA derived surface temperature estimates against 23 *in-situ* CMA stations. Both OL and FT DA derived estimates show relatively good agreement with the CMA surface temperature measurements in the average R statistics, where R_*OL*_ = R_*FTDA*_ ≈ 0.98, and the subscripts indicate estimates obtained from either OL or FT DA experiments. The good agreement in R between model and *in-situ* measurements demonstrate that the model is able to capture the day-to-day variability within the surface temperature time series. In addition, slight improvements are witnessed for FT DA where the average bias is reduced by 16% from −0.19 K (OL) to −0.16 K (FT DA), and the average RMSE is reduced by 2% from 3.04 K (OL) to 2.99 K (FT DA). The negative biases in the modeled surface temperature estimates might be explained by the negative biases in the MERRA-2 air temperatures, which had been reported by Xie et al. (2017). FT DA shows some improvements in bias and RMSE statistics relative to OL and also shows some tendency to correct the negative bias in the MERRA-2 air temperature. However, due to the relatively large variations of the computed statistics, no statistically significant skill differences (at a significance level of 5%) between OL and FT DA could be concluded here. There could exist several possibilities to explain the relatively insignificant improvement obtained from FT DA. First, the relatively insignificant improvements might be explained by the stations used during the evaluation since a single ground-based station is not able to represent the entire 1 by 1 km model grid cell. It might also be explained by the incorrect magnitudes of increments applied onto the FT DA state variable since we significantly simplify the error correlation between the surface temperature and the top-layer of soil temperature. Additional explanations might be the uncertainty in the assimilated freeze/thaw observations, which might arise from the simple interpolation strategy as discussed in section 2.2. Further, it is worthwhile pointing out that all CMA stations are installed in the eastern Tibetan Plateau and Taklamakan Desert, with relatively low elevations compared with the western Tibetan Plateau. Therefore, no solid conclusions could be made for FT DA performance in estimating surface temperatures at relatively high altitudes when compared against *in-situ* CMA measurements.

**FIGURE 6 |:**
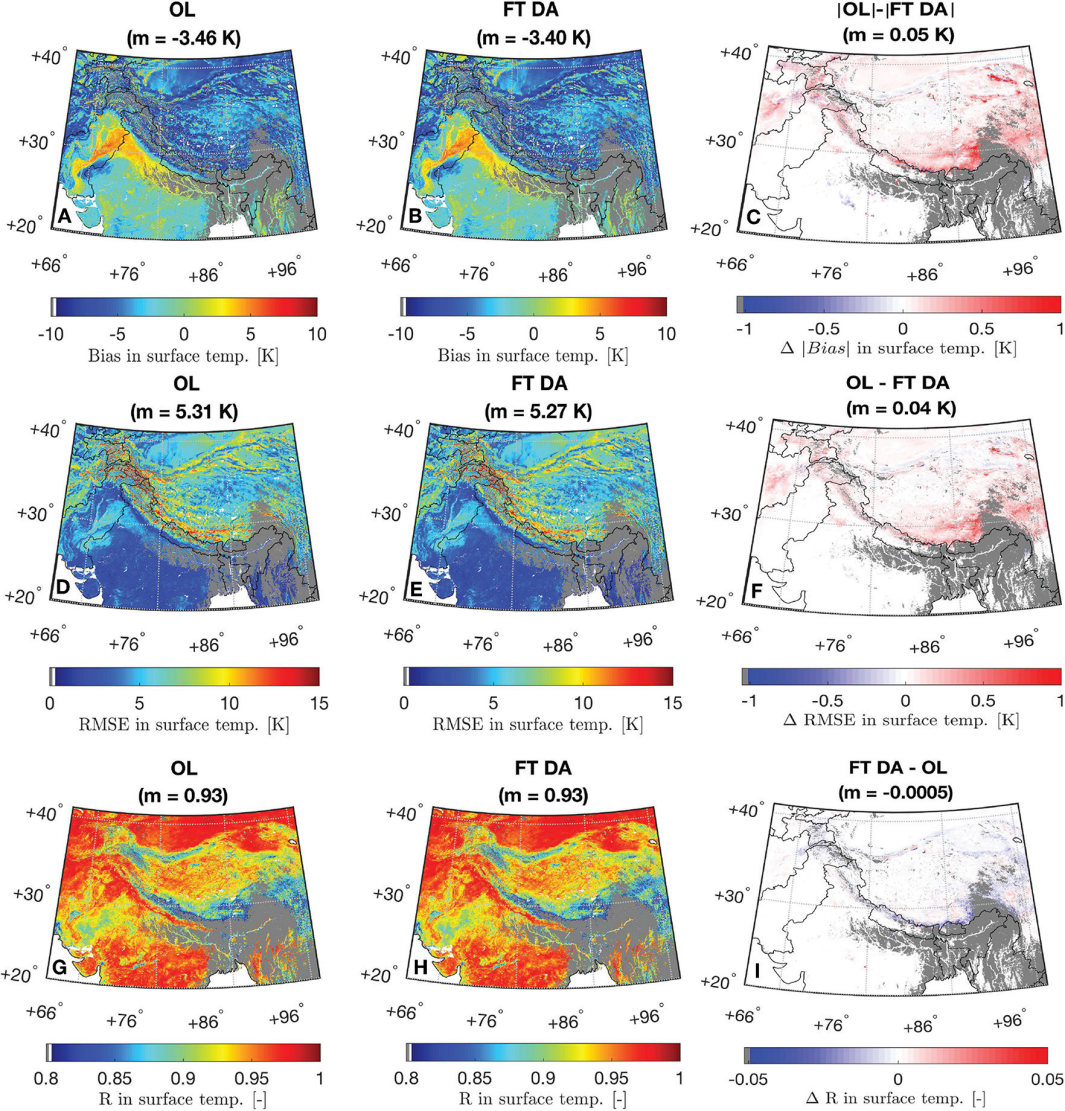
Spatial distribution of bias, RMSE, and R computed between daily-averaged **(A,D,G)** OL surface temperature and MODIS derived surface temperature, and **(B,E,H)** FT DA surface temperature and MODIS derived surface temperature. Spatial distribution of the change in the absolute value of bias (Δ|bias|) between OL and FT DA is shown in **(C)**. The |·| operator in the title denotes taking the absolute value of each corresponding bias. Spatial distributions of the change in the RMSE and in the R are shown in **(F,I)**, respectively. The red colors in **(C,F,I)** indicate FT DA agrees better with MODIS derived measurements than OL. Conversely, blue colors indicate that OL agrees better with MODIS. The title also demonstrates the spatial mean, m, computed for each map. Gray regions indicate grid cells with inadequate presence (i.e., <60 days) of MODIS derived daily-averaged measurements computed using Equation (4).

Figure 6 shows the spatial distributions of bias, RMSE, and R computed between daily-averaged OL, FT DA surface temperature estimates, and the MODIS derived surface temperature. Gray regions in Figure 6 indicate inadequate presence (i.e., <60 days) of MODIS derived daily-averaged surface temperature measurements computed using Equation (4), which are removed from all goodness-of-fit statistics computations. Noticeable positive biases in the surface temperature estimates are witnessed in Pakistan and Northern India along the Ganges and Indus rivers, covered with cropland (see Figure 1B). The area with the positive bias happens to be coincident with the “irrigated cropland” category as defined by the International Crops Research Institute for the Semi-Arid Tropics shown in http://geoagro.icarda.org/en/cms/metadata/index/762/SRT2-type%25252Bdrylands%25253A%25252Bland%25252Buse%25252Fland%25252Bcover. It is thus very likely that human-related irrigation activities introduce more evaporative cooling of the cropland, and the surface temperature drops accordingly. However, Noah-MP does not model irrigation-related activities, and therefore yields an overestimation of the surface temperature in this region. In addition, relatively high bias, high RMSE, and low R shown in Figure 6 are often found to be coincidental with glaciated area along the Pamir-Karakoram-Himalayas region shown in Olson and Rupper (2018), which are likely due to inaccurate model estimates since Noah-MP does not contain a glacier modeling routine. Future studies will be conducted to incorporate an advanced, hyper-resolution glacier model into the Noah-MP within LIS in order to better characterize model estimates along the glaciated region.

Figures 5, 6 also share some common findings. For example, both OL and FT DA yield negative, spatially-averaged bias in HMA possibly due to negative bias in the MERRA-2 air temperature. Relatively high, spatially-averaged correlation coefficients are shown for both OL and FT DA, where OL R ≈ DA R = 0.93. Slight improvements are witnessed for FT DA, where the average bias is reduced by 2% from −3.46 K (OL) to −3.40 K (FT DA) and the average RMSE is reduced by 1% from 5.31 K (OL) to 5.27 K (FT DA). Relatively large improvements obtained from FT DA relative to OL in terms of absolute bias and RMSE statistics are observed in the southern Tibetan Plateau, eastern Tibetan Plateau, and eastern Afghanistan compared with the rest of the region in HMA (see Figures 6C,F). However, due to the relatively large variations of the computed statistics, no statistically significant spatially-averaged skill differences (at a significance level of 5%) between OL and FT DA are observed. This might partly be attributed to the uncertainty in the MODIS/Terra and MODIS/Aqua surface temperature estimates (Zou et al., 2014) as well as the Equation (4) used to derive the daily-averaged surface temperature estimates. A more sophisticated semi-empirical model, different from the simple averaging method as applied in this study, for deriving daily-averaged surface temperatures based on MODIS/Terra and MODIS/Aqua is also provided by Zou et al. (2014). However, the method outlined in their study requires intensive measurements of the ground-based surface temperature to calibrate model-related coefficients (or parameters), which is not applicable in our study, and also out of the study scope.

**FIGURE 7 |:**
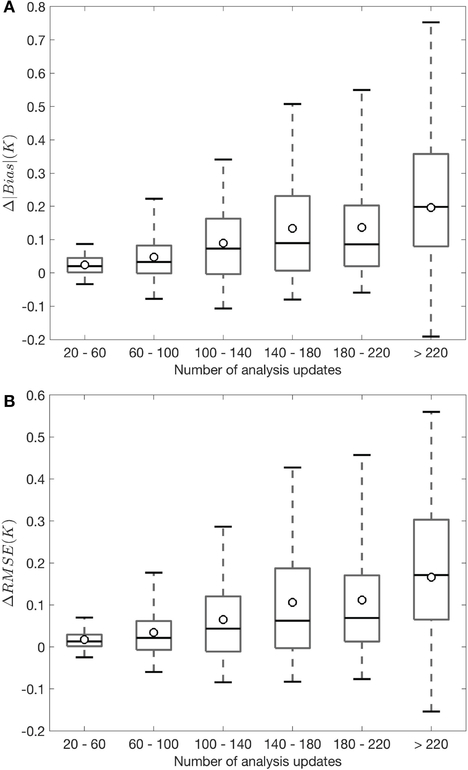
Box plots of **(A)** Δ|bias| and **(B)** ΔRMSE computed between OL and FT DA during the comparison against MODIS derived surface temperature, which are binned as a function of the number of analysis updates per grid cell. The boxes show the median (marked as the black line in the box) along with the 25th and 75th percentiles while the whiskers show the 5 and 95th percentiles. The spatially-averaged skill metrics are marked as dots for each bin. The positive ΔRMSE and positive Δ|bias| indicate skill improvements in the FT DA relative to OL.

Part of the reason for the statistically insignificant skill difference between OL and FT DA might also lie in the many zero-differences in bias and RMSE seen in Figure 6. For these zero-difference grid cells, seen across the majority of Pakistan, Southern India, and Western India, MEaSUREs observations and modeled freeze/thaw states always agree with each other, and hence no analysis updates take place. Therefore, the improvement (or degradation) magnitudes arising from FT DA are strongly dependent on the number of analysis updates that occurred during the assessment period. That is, Figure 7 further corroborates what is observed in Figures 6C,F. Figure 7A shows the box plots of change in the absolute value of bias (Δ|bias|) and Figure 7B shows the change in the RMSE (ΔRMSE) computed between OL and FT DA. The |·| operator denotes taking the absolute value of OL and FT DA bias. The change in the R (ΔR) does not show as box plots in Figure 7 because very little improvement (or degradation) is seen from Figure 6I. Figure 7 is binned as a function of the number of analysis updates per grid cell. The spatially-distributed number of updates per grid cell (N) throughout the assessment period are binned into six categories, including (1) 20 ≤ N ≤ 60, (2) 60 < N ≤ 100, (3) 100 < N ≤ 140, (4) 140 < N ≤ 180, (5) 180 < N ≤ 220, and (6) N > 220. The sample sizes (number of grid cells) for the six bins are 1112242, 1047492, 1135645, 765940, 125861, and 26369, respectively. As the number of analysis updates increases, there is generally a decreasing trend in the number of grid cells associated with each bin, especially when N > 140. This phenomenon is expected because of the relatively good agreement computed between satellite-based freeze/thaw observations and Noah-MP (model-only) simulated estimates (see Section 2.2). The positive ΔRMSE and positive Δ|bias| indicate skill improvements in the FT DA relative to OL. In general, the average skill improves with the number of analysis updates. It is encouraging to see that FT DA yields improvements of up to 0.58 K in RMSE and 0.77 K in the |bias| relative to OL during the comparison against MODIS-derived surface temperature estimates.

It is also important to analyze the effects of soil temperature estimates in response to the FT DA. Table 4 summarizes the goodness-of-fit statistics computed when comparing OL and FT DA derived soil temperature estimates against three *in-situ* CEOP stations along the Himalayas. Only the statistics for the top layer of soil (0–10 cm) and the second layer of soil (10–40 cm) are shown in Table 4 because no CEOP soil temperature measurements are available beyond 20 cm (see section 4.1). The total number of analysis updates for the colocated grid cells at Lukla station, Pyramid station, and Syangboche station are 172, 168, and 157, respectively. It is encouraging to see that the bias and the RMSE in the 0–10 cm soil temperature are reduced (on average) by 10 and 7%, respectively. The improvements in the top-layer of soil estimates also propagate through the deeper soil layers, where the bias and RMSE in the 10–40 cm soil temperature are reduced (on average) by 9 and 6%, respectively. However, slight degradations in R at both the top and second layers of soil are witnessed for FT DA relative to OL for the Syangboche station installed at (27.82°N, 86.72°E) covered with open shrub. Relatively poor R statistics are witnessed for FT DA or OL compared with the other two stations, especially for the second layer of soil. This is most likely due to the measurement gap (i.e., no measurements) seen between 4 November 2007 and 21 May 2008 in the CEOP Syangboche station (not shown). For example, the R computed between 1 September 2007 and 4 November 2007 comparing the CEOP top layer of soil and the CEOP second layer of soil temperature is 0.95, while the R computed between 22 May 2008 and 31 August 2008 is 0.40. Given the assumption that the measurement gap seen for Syangboche station arising from sensor failure, it is suspected that the soil temperature sensor had not been calibrated carefully after re-installation on 22 May 2008. However, no such information is documented by the website (or by the CEOP measurement flags), and therefore, all measurements remain as they are without implementing any additional quality control procedures other than the basic quality control activity mentioned in section 4.1.

Overall, model derived soil temperature estimates yield relatively large negative biases when compared against Lukla and Syangboche stations. The negative bias witnessed at Lukla station covered with open shrub is likely due to the negative bias of −5.03 K in the MERRA-2 air temperature, which is computed with respect to CEOP air temperature measurements. The negative bias observed at Syangboche station might be explained by (1) the sensor calibration issue discussed above, and/or (2) the positive bias of 0.37 kg/m^2^/h in the total precipitation. The overestimation of MERRA-2 total precipitation is mainly witnessed in June, July, August, and September when the air temperature is generally above freezing at Syangboche station. Rainfall infiltrates into the soil, and tends to cool the soil, which possibly leads to a negative bias in the model derived soil temperature profile. In addition, inaccurate model parameterization in the soil related properties, such as soil texture, soil layering, total soil depth, and soil organic carbon content might also negatively impact the model derived soil temperature estimates.

**FIGURE 8 |:**
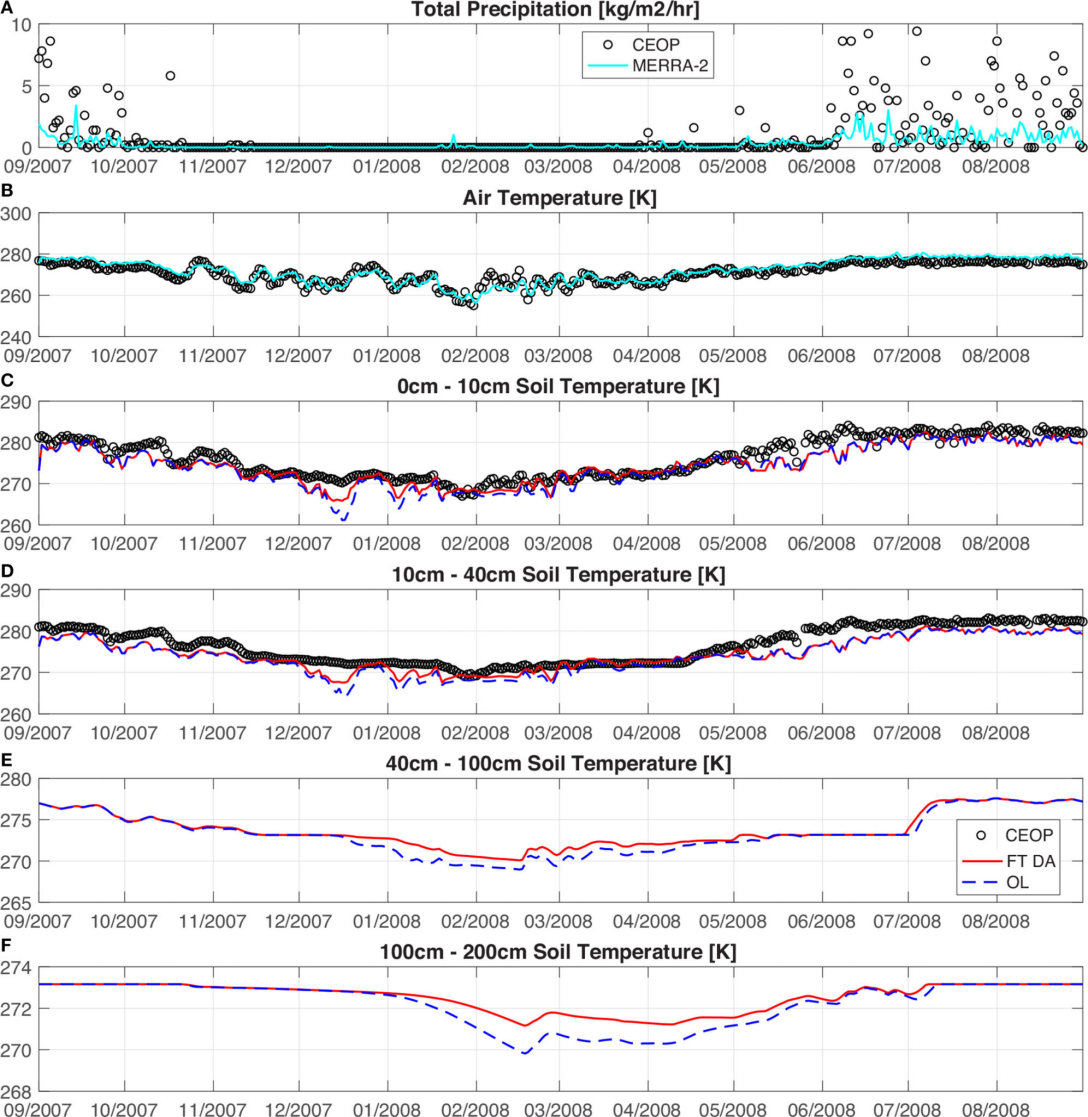
Comparisons of daily-averaged **(A)** MERRA-2 total precipitation and CEOP Syangboche station measured precipitation, and **(B)** MERRA-2 air temperature and CEOP Syangboche station measured air temperature. Comparisons of the model simulated, including both OL and FT DA derived, daily-averaged soil temperature profiles against CEOP measurements, including **(C)** top-layer (0–10 cm), **(D)** second-layer (10–40 cm), **(E)** third-layer (40–100 cm), and **(F)** fourth layer (100–200 cm). No CEOP measurements are available at the third and fourth soil layers. The evaluation period is from 1 September 2007 to 31 August 2008.

The goodness-of-fit statistics computed from the CEOP Pyramid station are generally better than the other two stations. Figure 8 shows several example time series of MERRA-2 precipitation, MERRA-2 air temperature, OL derived soil temperature estimates, and DA derived soil temperature estimates when compared against measurements collected by the CEOP Pyramid station installed at (27.96°N, 86.82°E) covered with sparse vegetation (i.e., barren land cover). The Pyramid station is shown here because (1) there is no measurement data gap within the assessment period, and (2) the vegetation effect is at its minimum compared with the other two stations. Due to the relatively high thermal inertia of the soil (especially for deep soil), the soil within the top 40 cm experiences more variability in the temperature estimates, but less so for deeper soil layers. The increase in the time lag of such fluctuations is also observed as the soil depth getting deeper, as shown in Figure 8. In general, the bias in the model derived top layer of soil temperature is reduced by 26%, and the RMSE is reduced by 16% as a result of FT DA relative to OL. The bias in the model derived second layer of soil temperature is reduced by 21% and the RMSE is reduced by 15% as a result of FT DA. Compared with CEOP measured total precipitation, MERRA-2 precipitation has a negative bias of −0.86 kg/m^2^/h. The most significant difference between model simulation and *in-situ* CEOP measurements occurs around mid-December. Besides the occasional underestimation of air temperature during this period, it is hypothesized that Noah-MP might underestimate snow on the ground. Since snow cover acts as an effective insulator to protect the ground surface and the underlying soil from heat loss when the air temperature is below freezing, Noah-MP with less snow cover might presumably underestimate soil temperature in such cases. Without further detailed ground-based snow information obtained from CEOP or from other colocated stations, it is rather difficult to discern exactly the origin of the error.

## 6. CONCLUSIONS AND FUTURE DIRECTIONS

A hyper-resolution (1 km) land data assimilation configuration is developed within the NASA LIS using the Noah-MP forced by the MERRA-2. Two different sets of DA experiments are conducted from 2007 to 2008, including the SC DA and FT DA. Before conducting any assimilation experiments, the model spin-up analysis is first conducted in order to achieve a more stable initial condition of the model states. It is found that the Noah-MP derived surface temperature generally requires 3–5 years for spin-up, and the fourth layer of soil temperature requires 3–8 years for spin-up. Longer spin-up periods are often witnessed in the Tibetan Plateau due to the existence of temperature extremes.

The performance of the SC DA system is evaluated via comparisons with daily-averaged, qualified GlobSnow SWE estimates as well as available ground-based snow depth measurements. In the comparison against ground-based snow depth measurements, the majority of the stations (13 of 14) show slightly improved goodness-of-fit statistics as a result of the SC DA relative to OL. In the comparison against GlobSnow SWE estimates colocated with GlobSnow-provided weather stations, all (three out of three) of the grid cells demonstrate slightly improved bias and RMSE in SC DA relative to OL. It is important to note that only four of the 14 stations are statistically significant, due to the limited sample size and relatively high sample variance. The limited sample size is partly attributed to the limited ground-based stations available in the complex HMA region as well as the single-year evaluation period showed in this study.

The performance of the FT DA system is evaluated via comparisons with daily-averaged, MODIS derived surface temperature product. The average skill in FT DA improves with the number of analysis updates. FT DA yields improvements of up to 0.58 K in RMSE and 0.77 K in the absolute bias relative to OL. In addition, slight improvements in bias and RMSE are also observed in the FT DA derived 0–10 and 10 cm–40 cm soil temperature estimates when compared to ground-based CEOP stations. That is, in the comparison against three ground-based soil temperature measurements along the Himalayas, the bias and the RMSE in the 0–10 cm soil temperature are reduced (on average) by 10 and 7%, respectively. The improvements in the top-layer of soil estimates also propagate through the deeper soil layers, where the bias and RMSE in the 10–40 cm soil temperature are reduced (on average) by 9 and 6%, respectively. In addition, in the comparison against 23 *in-situ* CMA stations, slight (but not statistically significant) improvements in RMSE and bias are both achieved as a result of the FT DA relative to OL at regions with relatively low elevations.

Some limitations associated with the SC DA and FT DA systems along with their evaluation strategies are also discussed. For example, the station representativeness issue persists among all *in-situ* measurements. It is relatively difficult to justify that a single ground-based station can represent the condition of a relatively large model grid cell, especially in the context of the complex terrain across HMA. Similarly, satellite-based snow products and surface temperature products are also prone to uncertainties. It is relatively difficult to discern the model uncertainty from the uncertainty embedded in the reference products used during the evaluation procedure. In addition, the SWE and snow depth increments, either positive or negative, as applied during the SC DA is somewhat subjective. The error correlation analyzed between modeled surface temperature and the top layer of soil temperature during the FT DA update process is overly simplified. Therefore, the increment magnitudes applied in both SC DA and FT DA systems (with DI methods) might be used as first-order adjustments or updates. In order to apply more accurate increments in both systems during the analysis update procedure, more sophisticated DA techniques, such as an ensemble Kalman filter, should be employed along with advanced, satellite-based, continuous remote sensing products at relatively fine spatial resolution. Furthermore, the soil parameterization, such as the total soil depth of 2 m with four layers in the current Noah-MP configuration, might not be deep enough to simulate the near-surface soil conditions accurately, especially in the cold regions (Lawrence et al., 2008; Sapriza-Azuri et al., 2018).

Despite the limitations discussed above, the two proposed DA schemes did show some promise in improving the predictability of SWE, snow depth, surface temperature, and soil temperature states across HMA. Future studies will be conducted to develop a multi-variate DA framework by integrating both SC DA and FT DA systems together. In addition, an improved meteorological forcing input, a glacier model, and a river routing routine would be useful to be included in the Noah-MP model to evaluate runoff in the region. The methods to generate improved forcings include, but are not limited to, (1) an advanced forcing downscaling framework (https://eospso.gsfc.nasa.gov/sites/default/files/eo_pdfs/Mar_Apr_2018_color%20508_0.pdf), (2) a meteorological forcing scaling framework (e.g., Voegeli et al., 2016), or (3) an ensemble-based bias correction framework when intensive ground-based snow observations are made available (e.g., Winstral et al., 2019). The runoff evaluation analysis will be beneficial to show whether the slight improvements seen in snow mass as a result of the SC DA would translate into runoff. Furthermore, more soil profile configurations should be carefully analyzed to assess their impacts on the soil temperature and moisture estimates in HMA. A combination of various snow DA techniques (i.e., by combining the DI method outlined in this study with an ensemble Kalman filter outlined in Xue et al., 2018) will also be studied in the future to better characterize SWE and snow depth estimates in HMA. Therefore, the DI-based DA scheme presented in this study can be used as a benchmark for evaluating more advanced DA schemes.

## Figures and Tables

**TABLE 1:** | Noah-MP model runtime options and parameters Q6 used in this study.

**Option**	**Value**	**Definition**

Vegetation model option	2	dynamic vegetation
Canopy stomatal resistance option	1	Ball-Berry type (Ball et al., 1987)
Soil moisture factor for stomatal resistance option	1	original Noah (Chen and Dudhia, 2001)
Runoff and groundwater option	1	TOPMODEL with groundwater (Niu et al., 2007)
Surface layer drag coefficient option	1	Monin-Obukhov (Brutsaert, 1982)
Supercooled liquid water option	1	No iteration (Niu and Yang, 2006)
Frozen soil permeability option	1	linear effects, more permeable (Niu and Yang, 2006)
Radiation transfer option	1	modified two-stream
Snow surface albedo option	2	CLASS (Verseghy, 1991)
Rainfall and snowfall option	1	Jordan (Jordan, 1991)
Lower boundary of soil temperature option	2	Noah (read from file mentioned in section 2.1)
Snow and soil temperature time scheme	1	semi-implicit

**Parameter**	**Unit**	**Value**

Number of soil layers	[−]	4
Each soil layer thickness (from top to bottom)	m	0.1, 0.3, 0.6, 1.0
Number of snow layers	[−]	3
Soil color index	[−]	4: for medium dark color soil

**TABLE 2:** | Initial conditions of model prognostic variables used for Noah-MP model run.

Variable	Unit	Value

Initial soil temperature for each layer (from top to bottom)	K	288.0, 288.0, 288.0, 288.0
Initial soil moisture for each layer (from top to bottom)	m^3^/m^3^	0.2, 0.2, 0.2, 0.2
Initial canopy air temperature	K	288.0
Initial canopy air vapor pressure	K	261.7
Initial wetted or snowed fraction of canopy	[−]	0.0
Initial vegetation temperature	K	288.0
Initial ground temperature	K	288.0
Initial snowfall on the ground	mm/s	0.0
Initial snow height	m	0.0
Initial snow water equivalent	mm	0.0
Initial depth to water table	m	2.5
Initial water storage in aquifer	mm	4900.0
Initial water in aquifer and saturated soil	mm	4900.0
Initial lake water storage	mm	0.0
Initial leaf mass	g/m^2^	9.0
Initial mass of fine roots	g/m^2^	500.0
Initial stem mass	g/m^2^	3.33
Initial mass of wood including woody roots	g/m^2^	500.0
Initial leaf area index	[−]	0.5
Initial stem area index	[−]	0.1
Initial momentum drag coefficient	[−]	0.0
Initial sensible heat exchange coefficient	[−]	0.0
Initial snow aging term	[−]	0.0
Initial soil water content between bottom of the soil and water table	m^3^/m^3^	0.0
Initial reference height of temperature and humidity	m	6.0

**TABLE 3:** | Statistics computed when comparing model derived, including both OL and SC DA derived, snow depth or SWE estimates against measurements obtained from GSOD, CHARIS, or GlobSnow.

Evaluation source - state, with station elevation (m)	Latitude (°)	Longitude (°)	Bias (cm)	OL RMSE (cm)	R	Bias (cm)	SC DA RMSE (cm)	R

CHARIS-snow depth, 2563	39.45	70.20	88.89	90.45	0.62	88.74	90.30	0.62
CHARIS-snow depth, 2234	39.44	69.66	63.35	66.66	0.70	**51.40**	**55.44**	0.70
CHARIS-snow depth, 1016	39.51	67.60	−6.44	8.08	0.61	−6.39	8.02	0.61
GSOD-snow depth, 345	40.12	67.84	1.24	3.03	0.70	0.99	2.92	0.76
GSOD-snow depth, 264	40.82	68.69	−7.14	9.41	0.11	−7.09	9.32	0.11
GSOD-snow depth, 474	40.98	71.59	−5.75	6.46	−0.09	−5.52	6.24	**0.06**
GSOD-snow depth, 765	40.92	72.95	2.02	8.00	0.23	1.97	8.00	0.23
GSOD-snow depth, 868	40.70	72.90	5.94	10.36	−0.03	5.84	10.34	−0.03
GSOD-snow depth, 604	40.37	71.76	−4.87	5.42	−0.13	−4.73	5.26	−0.11
GSOD-snow depth, 678	39.71	66.99	0.30	3.87	0.74	0.44	3.86	0.74
GSOD-snow depth, 2561	39.45	70.20	70.63	73.60	0.55	70.51	73.47	0.55
*GSOD-snow depth, 785	38.55	68.83	60.15	61.63	−0.05	**30.03**	**30.75**	**0.16**
GSOD-snow depth, 265	37.52	66.03	−3.48	4.69	0.85	−3.41	4.66	0.85
GSOD-snow depth, 2077	37.51	71.51	85.67	86.56	0.87	**77.06**	**78.27**	0.87
*GlobSnow-SWE	39.48	76.00	−0.30	1.10	0.62	−0.19	0.99	0.70
GlobSnow-SWE	39.77	78.56	−0.19	0.79	0.70	−0.12	0.74	0.69
GlobSnow-SWE	40.51	81.05	−0.19	0.93	0.73	−0.08	0.88	0.65

OL or SC DA derived estimates with statistically significant (at a significance level of 5%) metrics (i.e., bias, RMSE, and R) are bolded. The grid cell marked with an asterisk also provides time series in Figure 2. It is important to note that GlobSnow product only provides station coordinates, and no station elevation information is provided (see section 4.2).

**TABLE 4:** | Statistics computed when comparing model derived, including both OL and FT DA derived, top-layer (0–10 cm) and second-layer (10–40 cm) of soil temperature estimates against measurements obtained from CEOP.

Evaluation source-state	CEOP station name, with station elevation (m)	Bias (K)	OL RMSE (K)	R	Bias (K)	FT DA RMSE (K)	R

CEOP-top-layer of soil temp.	Lukla, 2660	−8.43	8.59	0.92	−8.17	8.36	0.92
*CEOP-top-layer of soil temp.	Pyramid, 5035	−2.07	2.79	0.92	**−1.54**	**2.34**	0.93
CEOP-top-layer of soil temp.	Syangboche, 3833	−5.15	5.47	0.75	−5.10	5.42	0.74
CEOP-second-layer of soil temp.	Lukla, 2660	−8.23	8.31	0.95	**−7.98**	**8.05**	0.95
*CEOP-second-layer of soil temp.	Pyramid, 5035	−2.50	2.89	0.94	**−1.98**	**2.48**	0.94
CEOP-second-layer of soil temp	Syangboche, 3833	−4.03	5.78	0.24	−3.95	5.74	0.21

OL or FT DA derived estimates with statistically significant (at a significance level of 5%) metrics (i.e., bias, RMSE, and R) are bolded. The grid cell marked with an asterisk also provides time series in Figure 8.

## Abbreviations:

AMSR: Advanced Microwave Scanning Radiometer
CEOP: Coordinated Enhanced Observing Period
CHARIS: Contribution to High Asia Runoff from Ice and Snow
CMA: Chinese Meteorological Administration
DA: data assimilation
DI: direct insertion
FT DA: freeze/thaw assimilation
GlobSnow: Global Snow Monitoring for Climate Research
GSOD: Global Summary of the Day
HMA: High Mountain Asia
LDAS: land data assimilation system
LDT: Land surface Data Toolkit
LIS: Land Information System
MEaSUREs: Making Earth System Data Records for Use in Research Environments
MERRA: Modern-Era Retrospective analysis for Research and Applications
MODIS: Moderate Resolution Imaging Spectroradiometer
Noah-MP: Noah multi-parametrization land surface model
OL: open-loop
SC DA: snow cover assimilation
SWE: snow water equivalent

## References

[R1] Andreadis KM, and Lettenmaier DP (2006). Assimilating remotely sensed snow observations into a macroscale hydrology model. Adv. Water Resour. 29, 872–886. doi: 10.1016/j.advwatres.2005.08.004

[R2] Arsenault KR, Houser PR, De Lannoy GJ, and Dirmeyer PA (2013). Impacts of snow cover fraction data assimilation on modeled energy and moisture budgets. J. Geophys. Res. Atmos 118, 7489–7504. doi: 10.1002/jgrd.50542

[R3] Arsenault KR, Kumar SV, Geiger JV, Wang S, Kemp E, Mocko DM, (2018). The land surface data toolkit (ldt v7.2) – a data fusion environment for land data assimilation systems. Geosci. Model Dev. 11, 3605–3621. doi: 10.5194/gmd-11-3605-2018

[R4] Ball JT, Woodrow IE, and Berry JA (1987). “A model predicting stomatal conductance and its contribution to the control of photosynthesis under different environmental conditions,” in Progress in Photosynthesis Research (Springer), 221–224.

[R5] Barnett TP, Adam JC, and Lettenmaier DP (2005). Potential impacts of a warming climate on water availability in snow-dominated regions. Nature 438:303. doi: 10.1038/nature04141 16292301

[R6] Bartsch A, Kidd RA, Wagner W, and Bartalis Z (2007). Temporal and spatial variability of the beginning and end of daily spring freeze/thaw cycles derived from scatterometer data. Remote Sens. Environ. 106, 360–374. doi: 10.1016/j.rse.2006.09.004

[R7] Bateni SM, Huang C, Margulis SA, Podest E, and McDonald K (2013). Feasibility of characterizing snowpack and the freeze–thaw state of underlying soil using multifrequency active/passive microwave data. IEEE Trans. Geosci. Remote Sens. 51, 4085–4102. doi: 10.1109/TGRS.2012.2229466

[R8] Brutsaert W (1982). Evaporation into the Atmosphere: Theory, History and Applications.

[R9] Cai X, Yang Z-L, David CH, Niu G-Y, and Rodell M (2014). Hydrological evaluation of the noah-mp land surface model for the mississippi river basin. J. Geophys. Res. Atmos. 119, 23–38. doi: 10.1002/2013JD020792

[R10] Cai X, Yang Z-L, Fisher JB, Zhang X, Barlage M, and Chen F (2016). Integration of Nitrogen Dynamics into the Noah-mp Land Surface Model v1. 1 for Climate and Environmental Predictions. Technical report, Pacific Northwest National Lab(PNNL), Richland, WA.

[R11] Chen F, Barlage M, Tewari M, Rasmussen R, Jin J, Lettenmaier D, (2014). Modeling seasonal snowpack evolution in the complex terrain and forested colorado headwaters region: a model intercomparison study. J. Geophys. Res. Atmos. 119, 13–795. doi: 10.1002/2014JD022167

[R12] Chen F, and Dudhia J (2001). Coupling an advanced land surface–hydrology model with the penn state–ncar mm5 modeling system. part i: Model implementation and sensitivity. Mon. Weather Rev. 129, 569–585. doi: 10.1175/1520-0493(2001)129<0569:CAALSH>2.0.CO;2

[R13] Colliander A, McDonald K, Zimmermann R, Schroeder R, Kimball JS, and Njoku EG (2012). Application of quikscat backscatter to smap validation planning: freeze/thaw state over alectra sites in alaska from 2000 to 2007. IEEE Trans. Geosci. Remote Sens. 50:461. doi: 10.1109/TGRS.2011.2174368

[R14] Crago RD, and Qualls RJ (2014). Use of land surface temperature to estimate surface energy fluxes: contributions of wilfried brutsaert and collaborators. Water Resour. Res. 50, 3396–3408. doi: 10.1002/2013WR015223

[R15] Dai L, Che T, Wang J, and Zhang P (2012). Snow depth and snow water equivalent estimation from amsr-e data based on a priori snow characteristics in xinjiang, china. Remote Sens. Environ. 127, 14–29. doi: 10.1016/j.rse.2011.08.029

[R16] De Lannoy GJ, Reichle RH, Arsenault KR, Houser PR, Kumar S, Verhoest NE, (2012). Multiscale assimilation of advanced microwave scanning radiometer–eos snow water equivalent and moderate resolution imaging spectroradiometer snow cover fraction observations in northern colorado. Water Resour. Res. 48. doi: 10.1029/2011WR010588

[R17] Dickinson RE, Shaikh M, Bryant R, and Graumlich L (1998). Interactive canopies for a climate model. J. Clim. 11, 2823–2836. doi: 10.1175/1520-0442(1998)011<2823:ICFACM>2.0.CO;2

[R18] Farhadi L, Reichle RH, De Lannoy GJ, and Kimball JS (2015). Assimilation of freeze–thaw observations into the nasa catchment land surface model. J. Hydrometeorol. 16, 730–743. doi: 10.1175/JHM-D-14-0065.1

[R19] Foster J, Chang A, and Hall D (1997). Comparison of snow mass estimates from a prototype passive microwave snow algorithm, a revised algorithm and a snow depth climatology. Remote sens. Environ. 62, 132–142. doi: 10.1016/S0034-4257(97)00085-0

[R20] Gao Y, Li K, Chen F, Jiang Y, and Lu C (2015). Assessing and improving noah-mp land model simulations for the central tibetan plateau. J. Geophys. Res. Atmos. 120, 9258–9278. doi: 10.1002/2015JD023404

[R21] Gelaro R, McCarty W, Suárez MJ, Todling R, Molod A, Takacs L, (2017). The modern-era retrospective analysis for research and applications, version 2 (merra-2). J. Clim. 30, 5419–5454. doi: 10.1175/JCLI-D-16-0758.1 32020988 PMC6999672

[R22] Ghatak D, Zaitchik B, Kumar S, Matin M, Bajracharya B, Hain C, (2018). Influence of precipitation forcing uncertainty on hydrological simulations with the nasa south asia land data assimilation system. Hydrology 5:57. doi: 10.3390/hydrology5040057

[R23] Hall D, and Riggs G (2016). Modis/Terra Snow Cover Daily l3 Global 500m Grid, version 6. Boulder, CO: National Snow and Ice Data Center.

[R24] Holmes T, De Jeu R, Owe M, and Dolman A (2009). Land surface temperature from ka band (37 ghz) passive microwave observations. J. Geophys. Res. Atmos. 114. doi: 10.1029/2008JD010257

[R25] Immerzeel W, and Bierkens M (2012). Asia’s water balance. Nat. Geosci. 5:841. doi: 10.1038/ngeo1643

[R26] Immerzeel WW, Droogers P, De Jong S, and Bierkens M (2009). Large-scale monitoring of snow cover and runoff simulation in himalayan river basins using remote sensing. Remote Sens. Environ. 113, 40–49. doi: 10.1016/j.rse.2008.08.010

[R27] Immerzeel WW, Van Beek LP, and Bierkens MF (2010). Climate change will affect the asian water towers. Science 328, 1382–1385. doi: 10.1126/science.1183188 20538947

[R28] Jin M (2004). Analysis of land skin temperature using avhrr observations. Bull. Am. Meteorol. Soc. 85, 587–600. doi: 10.1175/BAMS-85-4-587

[R29] Jin M, Dickinson R, and Vogelmann A (1997). A comparison of ccm2–bats skin temperature and surface-air temperature with satellite and surface observations. J. Clim. 10, 1505–1524. doi: 10.1175/1520-0442(1997)010<1505:ACOCBS>2.0.CO;2

[R30] Jordan R (1991). A One-Dimensional Temperature Model for a Snow Cover, Spec. Rep. 91–16, Cold Reg. Res. and Eng. Lab, US Army Corps of Eng., Hanover, NH.

[R31] Kim Y, Kimball J, Glassy J, and McDonald K (2018). Measures Northern Hemisphere Polar Ease-Grid 2.0 Daily 6 km Land Freeze/Thaw Status from amsr-e and amsr2, Version 1. Boulder, CO: National Snow and Ice Data Center.

[R32] Kim Y, Kimball JS, Glassy J, and Du J (2017). An extended global earth system data record on daily landscape freeze–thaw status determined from satellite passive microwave remote sensing. Earth Syst. Sci. Data 9, 133–147. doi: 10.5194/essd-9-133-2017

[R33] Kim Y, Kimball JS, McDonald KC, and Glassy J (2011). Developing a global data record of daily landscape freeze/thaw status using satellite passive microwave remote sensing. IEEE Trans. Geosci. Remote Sens. 49, 949–960. doi: 10.1109/TGRS.2010.2070515

[R34] Kumar SV, Peters-Lidard CD, Tian Y, Houser PR, Geiger J, Olden S, (2006). Land information system: an interoperable framework for high resolution land surface modeling. Environ. Model. Softw. 21, 1402–1415. doi: 10.1016/j.envsoft.2005.07.004

[R35] Lawrence DM, Slater AG, Romanovsky VE, and Nicolsky DJ (2008). Sensitivity of a model projection of near-surface permafrost degradation to soil column depth and representation of soil organic matter. J. Geophys. Res. Earth Surface 113. doi: 10.1029/2007JF000883

[R36] Liu X, and Chen B (2000). Climatic warming in the tibetan plateau during recent decades. Int. J. Climatol. 20, 1729–1742. doi: 10.1002/1097-0088(20001130)20:14<1729::AID-JOC556>3.0.CO;2-Y

[R37] Liu Y, Peters-Lidard CD, Kumar S, Foster JL, Shaw M, Tian Y, (2013). Assimilating satellite-based snow depth and snow cover products for improving snow predictions in alaska. Adv. Water Resour. 54, 208–227. doi: 10.1016/j.advwatres.2013.02.005

[R38] Lu H, Koike T, Yang K, Hu Z, Xu X, Rasmy M, (2012). Improving land surface soil moisture and energy flux simulations over the tibetan plateau by the assimilation of the microwave remote sensing data and the gcm output into a land surface model. Int. J. Appl. Earth Observ. Geoinform. 17, 43–54. doi: 10.1016/j.jag.2011.09.006

[R39] Lundquist JD, Hughes M, Henn B, Gutmann ED, Livneh B, Dozier J, (2015). High-elevation precipitation patterns: using snow measurements to assess daily gridded datasets across the sierra nevada, california. J. Hydrometeorol. 16, 1773–1792. doi: 10.1175/JHM-D-15-0019.1

[R40] Ma N, Niu G-Y, Xia Y, Cai X, Zhang Y, Ma Y, (2017). A systematic evaluation of noah-mp in simulating land-atmosphere energy, water, and carbon exchanges over the continental united states. J. Geophys. Res. Atmos. 122, 12245–12268. doi: 10.1002/2017JD027597

[R41] Meng X, Wang H, Wu Y, Long A, Wang J, Shi C, (2017). Investigating spatiotemporal changes of the land-surface processes in xinjiang using high-resolution clm3. 5 and cldas: soil temperature. Sci. Rep. 7:13286. doi: 10.1038/s41598-017-10665-8 29038535 PMC5643526

[R42] Mitchell KE, Lohmann D, Houser PR, Wood EF, Schaake JC, Robock A, (2004). The multi-institution north american land data assimilation system (nldas): utilizing multiple gcip products and partners in a continental distributed hydrological modeling system. J. Geophys. Res. Atmos. 109. doi: 10.1029/2003JD003823

[R43] Niu G-Y, and Yang Z-L (2006). Effects of frozen soil on snowmelt runoff and soil water storage at a continental scale. J. Hydrometeorol. 7, 937–952. doi: 10.1175/JHM538.1

[R44] Niu G-Y, Yang Z-L, Dickinson RE, Gulden LE, and Su H (2007). Development of a simple groundwater model for use in climate models and evaluation with gravity recovery and climate experiment data. J. Geophys. Res. Atmos. 112. doi: 10.1029/2006JD007522

[R45] Niu G-Y, Yang Z-L, Mitchell KE, Chen F, Ek MB, Barlage M, (2011). The community noah land surface model with multiparameterization options (noah-mp): 1. model description and evaluation with local-scale measurements. J. Geophys. Res. Atmos. 116:D12109. doi: 10.1029/2010JD015139

[R46] Olson MH, and Rupper SB (2018). Impacts of topographic shading on direct solar radiation for valley glaciers in complex topography (in review). Cryosphere. doi: 10.5194/tc-2018-64

[R47] Pulliainen J (2006). Mapping of snow water equivalent and snow depth in boreal and sub-arctic zones by assimilating space-borne microwave radiometer data and ground-based observations. Remote Sens. Environ. 101, 257–269. doi: 10.1016/j.rse.2006.01.002

[R48] Pulliainen JT, and Grandell J (1999). HUT snow emission model and its applicability to snow water equivalent retrieval. IEEE Trans. Geosci. Remote Sens. 37, 1378–1390. doi: 10.1109/36.763302

[R49] Qiu J (2016). Trouble in tibet. Nature 529:142. doi: 10.1038/529142a 26762440

[R50] Rasmy M, Koike T, Boussetta S, Lu H, and Li X (2011). Development of a satellite land data assimilation system coupled with a mesoscale model in the tibetan plateau. IEEE Trans. Geosci. Remote Sens. 49, 2847–2862. doi: 10.1109/TGRS.2011.2112667

[R51] Reichle RH (2008). Data assimilation methods in the earth sciences. Adv. Water Resour. 31, 1411–1418. doi: 10.1016/j.advwatres.2008.01.001

[R52] Reichle RH, Koster RD, De Lannoy GJ, Forman BA, Liu Q, Mahanama SP, (2011). Assessment and enhancement of merra land surface hydrology estimates. J. Clim. 24, 6322–6338. doi: 10.1175/JCLI-D-10-05033.1

[R53] Reichle RH, Kumar SV, Mahanama SP, Koster RD, and Liu Q (2010). Assimilation of satellite-derived skin temperature observations into land surface models. J. Hydrometeorol. 11, 1103–1122. doi: 10.1175/2010JHM1262.1

[R54] Ren Y-Y, Ren G-Y, Sun X-B, Shrestha AB, You Q-L, Zhan Y-J, (2017). Observed changes in surface air temperature and precipitation in the hindu kush himalayan region over the last 100-plus years. Adv. Clim. Change Res. 8, 148–156. doi: 10.1016/j.accre.2017.08.001

[R55] Riggs GA, Hall DK, and Román MO (2017). Overview of nasa’s modis and visible infrared imaging radiometer suite (viirs) snow-cover earth system data records. Earth Syst. Sci. Data 9, 765–777. doi: 10.5194/essd-9-765-2017

[R56] Rodell M, and Houser P (2004). Updating a land surface model with modis-derived snow cover. J. Hydrometeorol. 5, 1064–1075. doi: 10.1175/JHM-395.1

[R57] Rodell M, Houser P, Berg A, and Famiglietti J (2005). Evaluation of 10 methods for initializing a land surface model. J. Hydrometeorol. 6, 146–155. doi: 10.1175/JHM414.1

[R58] Rodell M, Houser P, Jambor U, Gottschalck J, Mitchell K, Meng C-J, (2004). The global land data assimilation system. Bull. Am. Meteorol. Soc. 85, 381–394. doi: 10.1175/BAMS-85-3-381

[R59] Salzmann N, Nötzli J, Hauck C, Gruber S, Hoelzle M, and Haeberli W (2007). Ground surface temperature scenarios in complex high-mountain topography based on regional climate model results. J. Geophys. Res. Earth Surface 112. doi: 10.1029/2006JF000527

[R60] Sapriza-Azuri G, Gamazo P, Razavi S, and Wheater HS (2018). On the appropriate definition of soil profile configuration and initial conditions for land surface–hydrology models in cold regions. Hydrol. Earth Syst. Sci. 22, 3295–3309. doi: 10.5194/hess-22-3295-2018

[R61] Smith T, and Bookhagen B (2018). Changes in seasonal snow water equivalent distribution in high mountain asia (1987 to 2009). Sci. Adv. 4:e1701550. doi: 10.1126/sciadv.1701550 29349294 PMC5771697

[R62] Takala M, Luojus K, Pulliainen J, Derksen C, Lemmetyinen J, Kärnä JP, (2011). Estimating northern hemisphere snow water equivalent for climate research through assimilation of space-borne radiometer data and ground-based measurements. Remote Sens. Environ. 115, 3517–3529. doi: 10.1016/j.rse.2011.08.014

[R63] Tedesco M, and Narvekar PS (2010). Assessment of the nasa amsr-e swe product. IEEE J. Sel. Top. Appl. Earth Observ. Remote Sens. 3, 141–159. doi: 10.1109/JSTARS.2010.2040462

[R64] Toure AM, Rodell M, Yang Z-L, Beaudoing H, Kim E, Zhang Y, (2016). Evaluation of the snow simulations from the community land model, version 4 (clm4). J. Hydrometeorol. 17, 153–170. doi: 10.1175/JHM-D-14-0165.1

[R65] Verseghy DL (1991). Class–a canadian land surface scheme for gcms. i. soil model. Int. J. Climatol. 11, 111–133. doi: 10.1002/joc.3370110202

[R66] Voegeli C, Lehning M, Wever N, and Bavay M (2016). Scaling precipitation input to spatially distributed hydrological models by measured snow distribution. Front. Earth Sci. 4:108. doi: 10.3389/feart.2016.00108

[R67] Walker JP, Houser PR, and Reichle RH (2003). New technologies require advances in hydrologic data assimilation. EOS Trans. Am. Geophys. Union 84, 545–551. doi: 10.1029/2003EO490002

[R68] Wan Z, Hook SJ, and Hulley GC (2015). Modis/Terra Land Surface Temperature/Emissivity Daily l3 Global 1km Grid, Version 6. NASA EOSDIS LP DAAC.

[R69] Wan Z, and Li Z-L (1997). A physics-based algorithm for retrieving land-surface emissivity and temperature from eos/modis data. IEEE Trans. Geosci. Remote Sens. 35, 980–996. doi: 10.1109/36.602541

[R70] Wang Z, Zeng X, and Decker M (2010). Improving snow processes in the noah land model. J. Geophys. Res. Atmos. 115. doi: 10.1029/2009JD013761

[R71] Winstral A, Magnusson J, Schirmer M, and Jonas T (2019). The bias-detecting ensemble: a new and efficient technique for dynamically incorporating observations into physics-based, multilayer snow models. Water Resour. Res. 55, 613–631. doi: 10.1029/2018WR024521

[R72] Wu T, Zhao L, Li R, Wang Q, Xie C, and Pang Q (2013). Recent ground surface warming and its effects on permafrost on the central qinghai-tibet plateau. Int. J. Climatol. 33, 920–930. doi: 10.1002/joc.3479

[R73] Xie Z, Hu Z, Gu L, Sun G, Du Y, and Yan X (2017). Meteorological forcing datasets for blowing snow modeling on the tibetan plateau: evaluation and intercomparison. J. Hydrometeorol. 18, 2761–2780. doi: 10.1175/JHM-D-17-0075.1

[R74] Xiong C, Shi J, Cui Y, and Peng B (2017). Snowmelt pattern over high-mountain asia detected from active and passive microwave remote sensing. IEEE Geosci. Remote Sens. Lett. 14, 1096–1100. doi: 10.1109/LGRS.2017.2698448

[R75] Xu Z, Gong T, and Li J (2008). Decadal trend of climate in the tibetan plateau–regional temperature and precipitation. Hydrol. Process. Int. J. 22, 3056–3065. doi: 10.1002/hyp.6892

[R76] Xue Y, Forman BA, and Reichle RH (2018). Estimating snow mass in north america through assimilation of amsr-e brightness temperature observations using the catchment land surface model and support vector machines. Water Resour. Res.54, 5879–7108. doi: 10.1029/2017WR022219 PMC6235457 30449910

[R77] Yang K, Wu H, Qin J, Lin C, Tang W, and Chen Y (2014). Recent climate changes over the tibetan plateau and their impacts on energy and water cycle: a review. Global Planet. Change 112, 79–91. doi: 10.1016/j.gloplacha.2013.12.001

[R78] Yang Z-L, and Niu G-Y (2003). The versatile integrator of surface and atmosphere processes: part 1. model description. Global Planet. Change 38, 175–189. doi: 10.1016/S0921-8181(03)00028-6

[R79] Yang Z-L, Niu G-Y, Mitchell KE, Chen F, Ek MB, Barlage M, (2011). The community noah land surface model with multiparameterization options (noah-mp): 2. evaluation over global river basins. J. Geophys. Res. Atmos. 116. doi: 10.1029/2010JD015140

[R80] Zaitchik BF, and Rodell M (2009). Forward-looking assimilation of modis-derived snow-covered area into a land surface model. J. Hydrometeorol. 10, 130–148. doi: 10.1175/2008JHM1042.1

[R81] Zhang G, Chen F, and Gan Y (2016). Assessing uncertainties in the noah-mp ensemble simulations of a cropland site during the tibet joint international cooperation program field campaign. J. Geophys. Res. Atmos. 121, 9576–9596. doi: 10.1002/2016JD024928

[R82] Zheng D, Van der Velde R, Su Z, Wen J, Booij MJ, Hoekstra AY, (2015). Under-canopy turbulence and root water uptake of a tibetan meadow ecosystem modeled by noah-mp. Water Resour. Res. 51, 5735–5755. doi: 10.1002/2015WR017115

[R83] Zou D, Zhao L, Wu T, Wu X, Pang Q, and Wang Z (2014). Modeling ground surface temperature by means of remote sensing data in high-altitude areas: test in the central tibetan plateau with application of moderate-resolution imaging spectroradiometer terra/aqua land surface temperature and ground-based infrared radiometer. J. Appl. Remote Sens. 8:083516. doi: 10.1117/1.JRS.8.083516

